# Odd-even conductance oscillations in *meta*-cycloparaphenylenes

**DOI:** 10.1126/sciadv.aeb8037

**Published:** 2026-03-04

**Authors:** Xuwei Song, Jia-Nan Gao, Kai Song, Chengjia Jing, Bingchen Liu, Mingliang Zhang, Junfeng Lin, Jing-Tao Lü, Yongfeng Wang, Huan Cong, Daoben Zhu, Yaping Zang

**Affiliations:** ^1^ Beijing National Laboratory for Molecular Sciences, CAS Key Laboratory of Organic Solids, Institute of Chemistry, Chinese Academy of Sciences, Beijing 100190, China.; ^2^ University of Chinese Academy of Sciences, Beijing 100049, China.; ^3^ Key Laboratory of Photochemical Conversion and Optoelectronic Materials, Technical Institute of Physics and Chemistry, Chinese Academy of Sciences, Beijing 100190, China.; ^4^ School of Physics and Wuhan National High Magnetic Field Center, Huazhong University of Science and Technology, Wuhan 430074, China.; ^5^ Wuhan Institute of Quantum Technology, Wuhan 430074, China.; ^6^ Center for Carbon-Based Electronics and Key Laboratory for the Physics and Chemistry of Nanodevices, Department of Electronics, Peking University, Beijing 100871, China.

## Abstract

Molecular-scale electronics seeks to transcend classical device paradigms by leveraging the quantum nature of charge transport. Molecular orbitals, as electron wave functions, exhibit spatially structured amplitude and nodal patterns that shape electron transmission. Yet, the spatial characteristics are difficult to resolve experimentally in single-molecule junctions. Here, we report the direct observation of length-dependent odd-even conductance oscillations that arise from sampling different regions of a single π-orbital at room temperature. This is enabled by anchor-free single-molecule junctions, where cyclic carbon nanohoop molecules form Au-π contacts with gold electrodes, allowing the intrinsic π-orbital profile to be probed directly. This minimal-contact design preserves orbital symmetry and reveals conductance variations linked to the orbital’s spatial amplitude distribution, as corroborated by first-principles transport calculations. These results demonstrate that the spatial structure of an individual molecular orbital can measurably influence room-temperature charge transport, providing a clear framework for understanding orbital contributions in molecular-scale electronic systems.

## Teaser

Carbon nanohoops show how the spatial structure of a single molecular orbital affects electronic conductance.

## INTRODUCTION

**Fig. 1.**
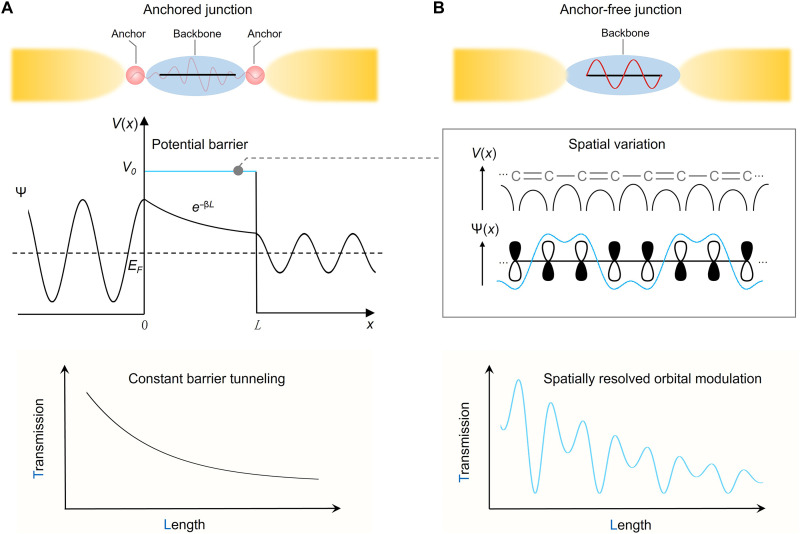
Effect of molecular orbital spatial distribution on the length-dependent transmission. (**A**) Schematic of an anchored single-molecule junction. In the constant-barrier tunneling model, the molecular backbone is treated as a spatially uniform potential barrier. The electron propagates as a plane wave in the electrodes and decays exponentially within the junction, resulting in a smooth, monotonic decrease in transmission with increasing molecular length. This model neglects any internal electronic structure of the molecule. (**B**) In real molecular systems, the atomic structure of the backbone introduces a nonuniform potential landscape. The resulting molecular orbitals exhibit spatially varying amplitude along the molecular axis. For example, alternating single and double C─C bonds give rise to a quasi-periodic potential and nodal patterns in the wavefunction. When the transport path is sampled with sufficiently fine resolution, the length-dependent transmission reveals oscillations that reflect the spatial structure of the molecular orbital, rather than a simple exponential decay.

Molecular electronics investigates charge transport through individual molecules, offering an atomically precise platform to study quantum phenomena and explore device functions beyond classical limits (*1*–*4*). The standard architecture for such studies is the single metal-molecule-metal junction (SMJ), where a molecule bridges two metallic electrodes via anchoring groups such as thiols or amines (*5*–*9*) (Fig. 1A). Although multiple molecular orbitals can contribute to charge transport, length-dependent tunneling in many SMJs is often interpreted within a simplified single-level framework (*10*, *11*). In this model, the molecule is approximated as a spatially uniform energy barrier: Its height is defined by the energy offset between the electrode Fermi level and the frontier orbital, and its width corresponds to the molecular length (*12*–*15*). As in the textbook square-barrier case (Fig. 1A), this model predicts an exponential decay of conductance with increasing molecular length, a trend consistently observed across various molecular oligomers with repeating units (*16*–*18*).

However, this simplified perspective neglects the spatial structure of molecular wavefunctions, including nodal planes, amplitude distribution, and phase coherence, all of which can profoundly affect electron transmission (Fig. 1B). As a prototypical example, quantum interference (QI) among different molecular orbitals gives rise to constructive or destructive wave behavior depending on the relative phase of the orbitals (*12*, *19*–*24*). A prior study demonstrated the role of nodal structure in modulating conductance via mechanically controlled interference in π-stacked dimers (*25*). There, conductance changes emerged from variations in the spatial overlap between frontier orbitals of two stacked molecules, effectively switching destructive interference on or off.

Unlike multiorbital interference, directly resolving the contribution of a single orbital’s spatial distribution to transport remains experimentally challenging. Figure 1B illustrates how the orbital amplitude varies along the molecular axis. When the transport path is sampled with sufficient resolution, the length-dependent transmission is modulated by the orbital’s spatial distribution, leading to oscillations that reflect the orbital’s structure, rather than a simple exponential decay (fig. S1). The challenge in isolating a single orbital’s contribution arises from the resolution limits imposed by current chemical anchoring strategies: Anchoring groups inevitably hybridize with the molecular orbitals, perturbing their symmetry and constraining the contact geometry (*26*–*29*), which makes it difficult to resolve how spatial features of the orbital influence transport.

**Fig. 2.**
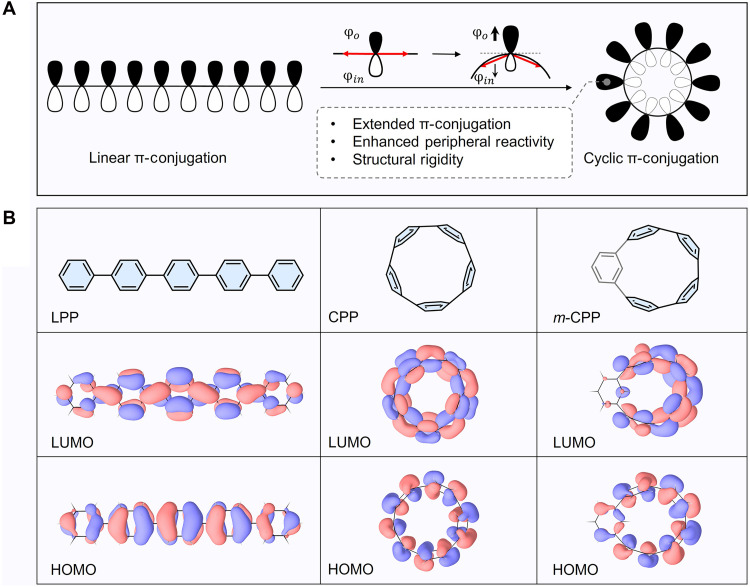
Comparison of linear and cyclic π-conjugated systems and their frontier orbital characteristics. (**A**) Linear π-conjugation versus cyclic π-conjugation. (**B**) Structural and frontier orbital comparisons of linear versus cyclic π-conjugated systems. In linear oligoparaphenylenes (LPPs), π-orbital overlap extends along a planar backbone. In contrast, carbon nanohoops formed by cyclically connected phenylene units exhibit curvature-induced orbital polarization, enhanced peripheral reactivity, and increased structural rigidity. Despite their distinct geometries, the LPPs, CPPs, and *m*CPPs all display frontier orbitals with similarly resolved nodal topologies.

**Fig. 3.**
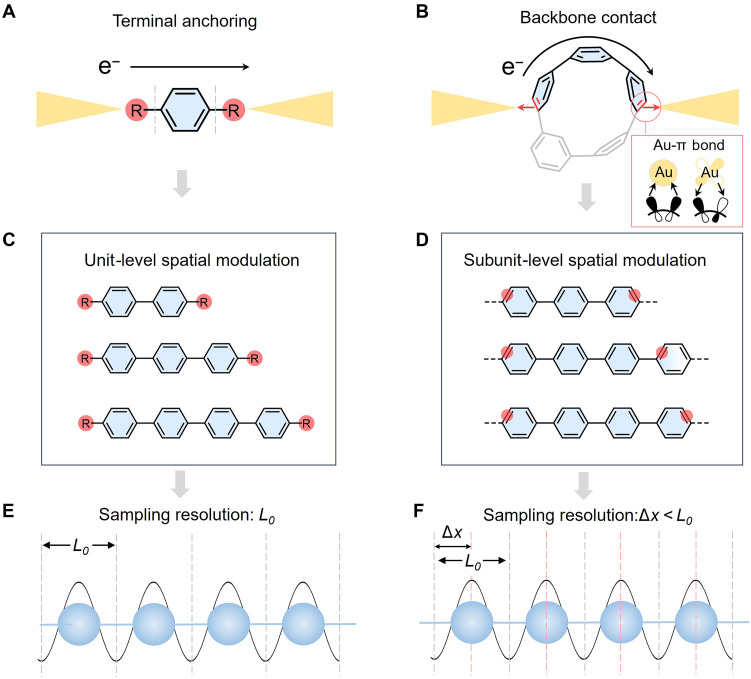
Comparison of anchoring and anchor-free strategies and their spatial resolution in single-molecule junctions. (**A**) Schematic of terminal anchoring, where the molecule connects to electrodes through predefined anchor groups (R) located at the termini of the linear molecular backbone. (**B**) Schematic of direct backbone contact in *m*[*n*]CPPs junctions, where gold electrodes form direct η^2^-type Au-π bonds with individual C=C bonds on the phenylene rings. This bonding involves electron donation from the π orbital of the curved phenylene ring to the Au 6s orbital and back-donation from the Au 5d orbital to the π* antibonding orbital of the ring. (**C**) Terminal anchoring enables unit-level spatial sampling, where conductance reflects an average over the full repeat units. (**D**) In contrast, the anchor-free backbone contact allows greater spatial resolution along the molecular backbone, capturing more localized electronic features. (**E**) Conceptual illustration of terminal anchoring, where spatial resolution is limited to the repeat unit length *L*_0_. (**F**) In the anchor-free configuration, contact can be tunned within Δ*x* < *L*_0_, revealing conductance modulations arising from spatial variations in the orbital amplitude within a single unit.

Here, we address these limitations by using carbon nanohoops—cycloparaphenylene molecules with curved π-conjugated frameworks first synthesized by Bertozzi and Jasti—as model systems for orbital-resolved transport studies (*30*–*33*) (Fig. 2). Their inherent curvature enables Au-π coupling directly to the carbon backbone, circumventing conventional terminal anchoring constraints (*34*–*37*) (Fig. 3). This anchor-free design allows structural modulation on a length scale finer than the orbital’s spatial modulation pattern, making it possible to resolve how different regions of a single orbital contribute to charge transport. By systematically varying nanohoop size, we observe odd-even conductance oscillations superimposed on the expected exponential decay, reflecting the nodal structure of the frontier molecular π-orbital as illustrated by density functional theory (DFT) calculations. These findings demonstrate that the spatial nodal pattern of a single molecular orbital can measurably influence charge transport at room temperature, providing a basis for understanding how orbital structure contributes to quantum transport in molecular-scale systems.

## RESULTS

Figure 2 shows the structures of the cycloparaphenylenes ([*n*]CPPs) and their *meta*-substituted counterparts (*m*[*n*]CPPs). This carbon nanohoops maintain classical π-conjugation while introducing well-defined geometric curvature, which leads to frontier orbitals with spatially well-defined nodal topologies, reminiscent of those in linear oligophenylenes. DFT calculations reveal that the highest occupied molecular orbital (HOMO) is predominantly localized on the individual phenylene rings, with nodal planes at the interunit connections. In contrast, the lowest unoccupied molecular orbital (LUMO) is delocalized across the bridging regions between rings (see fig. S2 for detailed orbital analysis). Both orbitals exhibit periodic modulation with a spatial period matching a single phenylene unit, establishing the nodal pattern that governs how orbital characteristics evolve along the backbone.

The curved geometry of the nanohoop further activates the peripheral π-orbitals, enabling direct η^2^-type Au-π bonding between gold electrodes and individual C=C bonds within the phenylene rings (*34*) (Fig. 3B and fig. S3). DFT calculations confirm that this bonding involves electron donation from molecular π orbitals to Au 6s orbitals, along with back-donation from Au 5d orbitals into π* orbitals (see more analysis in fig. S4). This carbon-backbone contact strategy eliminates the need for terminal anchoring groups (anchor-free architecture) and allows direct coupling to the intrinsic π-framework (fig. S5). At the same time, this architecture provides multiple possible Au-π binding sites (particularly in larger nanohoops), which necessitates careful analysis to identify the junction configurations relevant to charge transport.

The cyclic geometry naturally supports two parallel transport paths. Isolating a single, well-defined conduction channel is essential for resolving the influence of orbital spatial features on transport. To achieve this, a *meta*-substituted phenylene unit is introduced to break the molecular symmetry (*31*) and disrupt orbital delocalization along one path (Fig. 2). This selectively suppresses conduction through that route, as confirmed by transmission calculations (fig. S6), leaving a dominant transport pathway along the para-connected π-conjugated segment. With a single pathway established, varying the nanohoop size systematically shifts the electrode-binding positions along the backbone, allowing us to examine how local variations in orbital amplitude affect the measured conductance (Fig. 3, D and F).

**Fig. 4.**
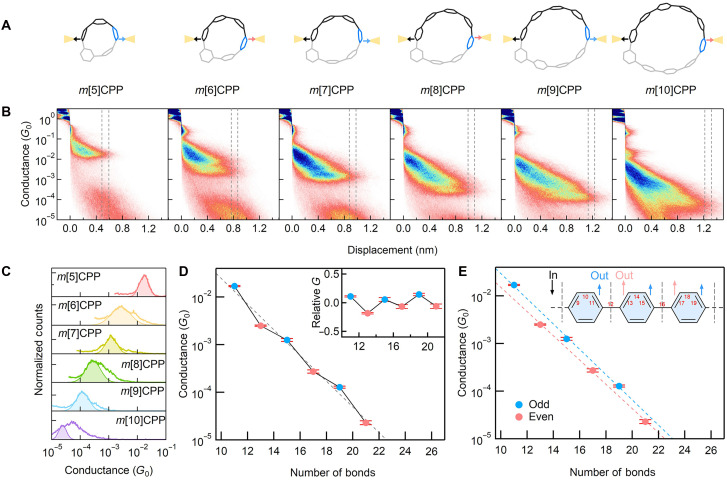
Geometry and conductance characteristics of *m*[*n*]CPPs SMJs. (**A**) Proposed anchor-free configurations of *m*[*n*]CPP SMJs, ranging from *m*[5]CPP to *m*[10]CPP. (**B**) Experimental 2D conductance-displacement histograms of *m*[*n*]CPP SMJs with different sizes. The vertical lines at the end of the molecular feature indicate the window for determining the conductance profiles presented in (C), defined as 85% of the maximum plateau extension distance. (**C**) 1D conductance distribution of *m*[*n*]CPPs molecules with different lengths determined from the profiles in the 2D histograms. (**D**) Single-molecule conductance of *m*[*n*]CPPs (*n* = 5 to 10) plotted against the number of bonds across the transport path [inset of (E)]. The overall trend follows an exponential decay with increasing junction length (gray dashed line), whereas a distinct odd-even oscillation is superimposed. Blue and red dots denote odd-membered and even-membered *m*[*n*]CPPs, respectively. Error bars represent the SD from five independent STM-BJ measurements, reflecting dataset-to-dataset reproducibility. Inset: Relative conductance values defined as *G*_rel_ = *G*_exp_ − *G*_fit_, where *G*_fit_ is the baseline exponential decay. The nearly constant amplitude of the oscillation across molecular lengths demonstrates the structural robustness of the parity-induced conductance modulation. (**E**) Both the even and odd groups follow the exponentially decay behavior exp(−β*N*) with identical β factor. Inset: Schematic of electron transport paths in the *m*CPP backbone. Electrons are injected at bond 1 (black arrow) and extracted from bonds labeled with blue (odd) or red (even) arrows (see more details in fig. S7).

To investigate how the spatial nodal structure of frontier orbitals modulates coherent tunneling transport, we synthesized a series of *m*[*n*]CPPs with ring sizes ranging from *n* = 5 to 10, following established protocols (*31*, *38*). Single-molecule conductance measurements were performed using the scanning tunneling microscopy–break junction (STM-BJ) technique (see Materials and Methods). Although multiple Au-π anchoring sites are available along the curved carbon backbone, junction rupture consistently occurs at the farthest-separated sites on the ring, as observed in our previous study (*34*). Because of the symmetry breaking introduced by the *meta*-linked units in *m*[*n*]CPPs, the longest junction configuration becomes geometrically well defined: One electrode binds at a *para*-phenylene position, while the other attaches to the adjacent *meta*-phenylene site (Fig. 4A). This structural asymmetry gives rise to two distinct classes of junctions depending on ring size. In odd-numbered *m*[*n*]CPPs, the second contact forms on the lower half of the ring, whereas in even-numbered rings, it shifts to the upper half. A detailed justification of these junction configurations based on binding-energy analysis, geometric evaluation, and STM-BJ length statistics is provided in a later section.

Experimental results are presented in two-dimensional (2D) conductance-displacement histograms (Fig. 4B) and corresponding 1D conductance histograms (figs. S8 and S9). Both histograms exhibit clear molecular conductance features for the entire series of *m*[*n*]CPPs molecules, with the plateau lengths increasing as ring size expands. The larger molecules also show increasingly sloped plateau distributions, consistent with the fact that a larger ring offers more possible Au-π binding sites (fig. S10). This trend supports the proposed Au-π binding mechanism and aligns with expected behavior in STM-BJ experiments, where short, high-conductance plateaus generally appear with the highest probability, particularly for cyclic systems such as *m*[*n*]CPPs with multiple electrode-molecule binding configurations. Many junctions break from these shorter configurations before reaching the fully extended geometry, resulting in lower occurrence probabilities for the longest plateaus in the 2D maps. For a consistent comparison of intrinsic length-dependent transport across the series, we therefore focus on the lowest-conductance, longest plateaus that occur just before rupture. Quantitative analysis of these plateaus matches closely with theoretical predictions (fig. S11), confirming the structural accuracy of our models. By profiling these fully extended configurations (defined as 85% of the maximum plateau extension distance), we extracted conductance values for *m*[*n*]CPP junctions (Fig. 4C), enabling a systematic study of their transport properties (detailed data analysis methods are provided in the Supplementary Materials).

To understand the observed conductance variations, we plotted the measured values against the through-bond transport distance, defined by the minimal number of C─C bonds (*N*) between the two anchoring sites. The resulting trend follows an overall exponential decay, *G* ∝ exp(−β*N*), characteristic of off-resonant tunneling. Superimposed on this decay, however, are distinct even-odd oscillations: Odd-numbered *m*[*n*]CPPs consistently exhibit higher conductance than even-numbered ones, despite having similar transport lengths (Fig. 4, D and E). This trend is highly reproducible across independent datasets (figs. S12 to S28), and the same oscillatory behavior is obtained whether conductance is extracted from 2D histograms (main analysis) or 1D histograms (fig. S9). The odd-even pattern also persists when conductance is plotted against the experimentally measured junction length or the DFT-derived through-space distance (figs. S29 to S32), confirming its robustness and intrinsic molecular origin.

This conductance alternation is absent in CPPs, as shown in our previous study (*34*), where the conductance decreases smoothly without oscillation (figs. S33 and S34). This contrast highlights that the *meta* substitution is required for the odd-even differences to emerge; in symmetric CPPs, the presence of two parallel transport pathways masks such variations (figs. S35 to S37). To further quantify this distinction, we fit exponential decay curves separately to the odd and even subsets. Both series yield nearly identical decay constants (β), comparable to those of CPPs, confirming that the tunneling attenuation is governed by the same oligophenylene backbone (fig. S34 and S38).

To elucidate the origin of these oscillations, we conducted first-principles calculations using DFT combined with the nonequilibrium Green’s function (NEGF) formalism (see “Theoretical methods” in Materials and Methods). Because multiple Au-π contact sites are, in principle, accessible on the nanohoop backbone, we first evaluated their relative stabilities. Binding-energy calculations reveal that Au preferentially forms η^2^(C=C) coordination at the double bond adjacent to an inter-ring single bond and that this interaction is strongly enhanced by molecular curvature. Consequently, only the curved *para*-phenylene units serve as viable binding sites, whereas the nearly planar *meta*-phenylene unit does not (figs. S39 and S40).

**Fig. 5.**
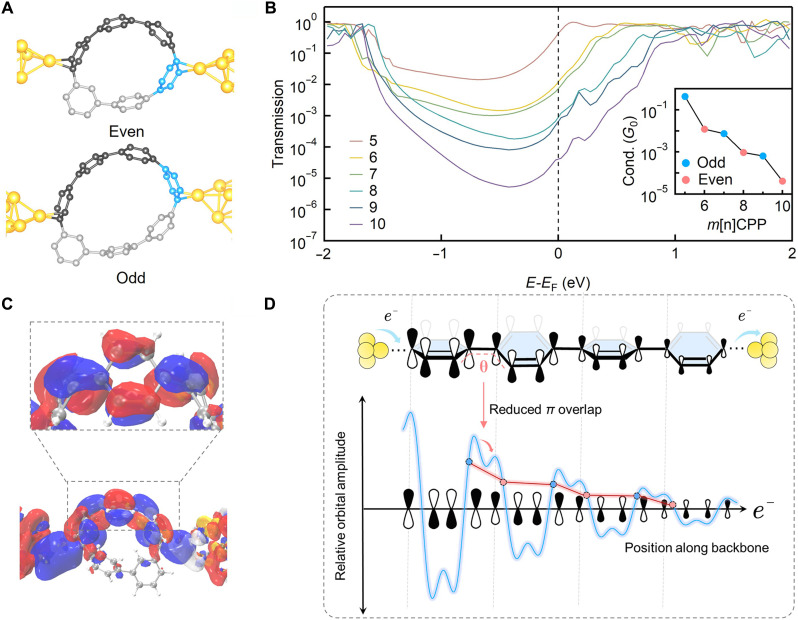
Theoretical explanation of the even-odd conductance oscillations. (**A**) Proposed contact configuration of *m*[*n*]CPPs SMJs with even (6) and odd (7) phenylene rings. (**B**) DFT-calculated transmission spectra of *m*[*n*]CPPs SMJs ranging from *m*[5]CPP to *m*[10]CPP. Inset: Dependence of calculated conductance at the *E*_F_ on the number of benzene rings. (**C**) Spatial distribution of the most dominant eigenchannel injected from left side and zoom-in plot of isosurface on the phenylene backbone. (**D**) Schematic illustration of how the spatial nodal pattern of the π orbital gives rise to the odd-even effect. The DFT-calculated eigenchannel of the dominant transport pathway shows that the π-orbital amplitude varies across both intra-ring and inter-ring positions, producing an oscillatory nodal pattern along the backbone. The blue curve represents a schematic 1D projection of this spatial modulation (“relative orbital amplitude”), and the red trace marks the local amplitudes sampled by the electrode binding sites in odd-numbered and even-numbered *m*CPPs. Because these two junction geometries probe different parts of the nodal pattern, odd and even members experience systematically different local orbital amplitudes at their contact points, giving rise to the observed odd-even conductance differences.

Geometric analysis of the fully extended configuration identifies the junction arrangement that maximizes the Au─Au separation, and STM-BJ plateau-length statistics confirm that this configuration is reproducibly observed in experiment (figs. S41 and S42). With this binding motif and extended geometry established, the junction structures across the *m*[*n*]CPPs series can be assigned: One electrode binds to the same phenylene unit in all members, whereas the second electrode binds to the lower side of the ring in odd-numbered *m*[*n*]CPPs and to the upper side in even-numbered ones (Fig. 5A).The corresponding transmission calculations reveal a clear parity-dependent pairing in both the LUMO energies and the transmission amplitudes (Fig. 5B and figs. S35, S43, and S44). When plotted against molecular size, the computed Fermi-level transmission exhibits a robust odd-even alternation that closely matches the experimental results (inset of Fig. 5B), confirming the parity effect.

To understand the origin of this parity dependence, we analyzed the dominant transmission eigenchannel of the junction (Fig. 5C and figs. S44 and S45). The calculations show that electrons travel mainly through a single para-connected pathway, and the spatial distribution of this eigenchannel follows the LUMO of the isolated molecule. Along this path, the transmission strength is not uniform: It is higher on phenylene rings and notably attenuated at the inter-ring C─C bonds, where torsion weakens π-overlap (fig. S45). As a result, length increments that introduce an additional inter-ring bond cause a large change in transmission, whereas changes confined within a phenylene unit have only a minor effect. Because the electrode-binding sites in odd and even members fall at different positions along this nonuniform transmission landscape, odd-numbered *m*[*n*]CPPs bind at regions of higher local transmission strength and therefore show higher conductance, whereas even-numbered members bind at lower-strength regions and exhibit reduced conductance. This difference in the sampled positions along the same single transport pathway provides a direct explanation for the observed parity-dependent conductance trend (fig. S46).

To further clarify this mechanism, we constructed a minimal schematic model that reflects the spatial variation of the frontier orbital along the backbone together with the expected exponential attenuation of off-resonant tunneling. Weighting each binding site by its local orbital amplitude reproduces the oscillatory conductance pattern, as illustrated schematically in Fig. 5D, where the spatial variation of the orbital along the backbone is reflected in the odd-even modulation. Last, to show that this effect does not depend on curvature-induced torsion of the nanohoop backbone, we calculated transmission spectra for planar oligophenylenes. The same oscillatory trend appears (figs. S47 and S48), demonstrating that the parity dependence arises from the intrinsic spatial nodal topology of the π-orbital, rather than from torsional distortions of the cyclic backbone.

Although conductance oscillations linked to parity effects have been reported in atomic chains, saturated molecular wires such as alkanes, silanes, and germanes, those effects typically arise from end-group orientation or edge-state modulations that alter molecular orbital character (*39*–*48*). In contrast, our system preserves a fixed orbital nodal topology across the entire series. The observed odd-even oscillations here arises from how different anchoring positions probe the spatial amplitude pattern of a single π-orbital. Although current techniques allow only discrete contact geometries to be accessed, future advances in controlling electrode binding sites may further enable systematic tuning of conductance through the spatial characteristics of molecular orbitals (fig. S49).

## DISCUSSION

In summary, we demonstrate the direct observation of conductance oscillations arising from the spatial structure of a single molecular orbital in single-molecule junctions, yielding a pronounced odd-even effect across the cyclic *m*CPPs series. This result reveals a distinct mechanism for tuning charge transport at the molecular scale. Collectively, these findings indicate that cyclic molecular architectures can be rationally engineered as functional components, such as molecular switches, offering versatile building blocks for future molecular electronics.

## MATERIALS AND METHODS

### Synthetic of *meta*-[*n*]cycloparaphenylenes (*m*[*n*]CPPs)

The series of *m*[*n*]CPPs with varied sizes (*n* = 5 to 10) were synthesized following previously reported procedures (*31*, *38*).

### STM-BJ measurements

For single molecule conductance measurements, 0.1 mM solution of *m*[*n*]CPPs (*n* = 5 to 10) in 1,2,4-trichlorobenzene (TCB) was prepared under ambient conditions at room temperature. Single-molecule junctions were formed and characterized using a custom-built STM-BJ setup with Au tips (99.999% purity) and Au-coated substrates as electrodes at an applied bias of 100 mV. Specifically, the Au tip is driven into and out of contact with the Au substrate coated with molecular solution. During the breaking of the Au─Au atomic contact, individual molecules were captured between the Au tip and Au-coated substrate. Current was continuously recorded during each junction formation, yielding conductance traces versus tip displacement. The conductance is determined from *G* = *I*/*V*. More than 5000 traces were collected without any data selection to construct both 1D and 2D conductance histograms.

### Theoretical methods

#### First-principles transport calculation

The geometry optimization for [*n*]CPPs and *m*[*n*]CPPs was performed using the Perdew-Burke-Ernzerhof (PBE) exchange-correlation functional, implemented by the Fritz Haber Institute ab initio molecular simulation (FHI-aims) package (*49*–*56*). The corresponding frontier molecular orbitals (HOMO and LUMO) presented in figs. S2 and S5 were obtained at the same level of theory.

The transmission spectra in figs. S6, S35, S36, S43, S44, S47, and S48 were obtained through combined DFT and NEGF (DFT-NEGF) calculations performed using the AITRANSS (ab initio transport simulations) code implemented within the FHI-aims framework. In this modeling approach, the electrodes were represented by finite gold clusters. Specifically, the molecular junction was initially constructed by attaching two four-atom Au clusters (with fixed Au─Au bond lengths of 2.88 Å) to both ends of the molecule for structural relaxation. Subsequently, the small clusters were replaced with 60-atom Au pyramidal electrodes (six layers) to better simulate the metallic contacts.

The transmission spectra and eigenchannels presented in Fig. 5 and fig. S45 were calculated using the SIESTA package (*57*) in combination with Inelastica code (*58*, *59*), using the DFT-NEGF method. In our simulations, the electrodes are modeled as semi-infinite Au(111) surfaces with a 5 × 5 Au atom cross section. The PBE exchange-correlation functional is applied consistently throughout the calculations. Double-ζ basis sets including polarization functions are used for C and H atoms, whereas single-ζ basis sets with polarization are applied to Au atoms. The energy cutoff is set to 150 rydbergs (Ry). The transmission spectra are averaged over 5 × 5 *k*-point in the Monkhorst-Pack scheme. The results of convergence tests are checked against basis set size, energy cutoff, and *k*-point sampling.

#### Strain energy calculation

The strain energy calculations were performed using Gaussian 16 (*60*) at the B3LYP/6-31G* level. The StrainViz program developed by Jasti and colleagues (*61*) is used for visualization. The results are presented in fig. S3.

#### Bond order calculation

The natural adaptive orbital (NAdO) analysis (*62*) was performed using Gaussian 16 at the B3LYP/6-311G** level for C and H atoms and B3LYP/SDD level for Au atoms, together with the Multiwfn package (*63*). Structure relaxation of the *m*[*n*]CPPs junction was carried out using a junction model where the *m*[*n*]CPP molecule is attached to two Au clusters, each containing four Au atoms with fixed Au─Au bond length of 2.88 Å. The results are presented in fig. S4.

#### DFTB simulations

To investigate the position-dependent charge transport characteristics of standard conjugated systems, we performed density functional–based tight binding (DFTB)–based calculations on a planar tetraphenyl with viable electrode anchoring sites (*64*–*66*) (fig. S49). The electrodes were modeled as single gold atoms, and the electrode-molecule coupling was treated within the wide-band approximation using a constant imaginary self-energy (−*i*Γ/2, Γ = 0.1 eV). This approach enabled the construction of an effective Hamiltonian that captures the essential features of the electrode-molecule interface. The resulting transmission spectra *T*(E) revealed pronounced oscillations in conductance at the Fermi energy (*E*_F_) as a function of electrode separation (i.e., conjugation length), consistent with our theoretical predictions.

## Supplementary Materials

### This PDF file includes:

Supplementary Text Figs. S1 to S49 References
