# Supplementary material for: Odd-even conductance oscillations in meta-cycloparaphenylenes
Source: Sci Adv. 2026 Mar 4;12(10):eaeb8037. doi: 10.1126/sciadv.aeb8037 (PMC12959390; doi:10.1126/sciadv.aeb8037)
Supplement: Supplementary file 1 — Supplementary Text Figs. S1 to S49 References [file sciadv.aeb8037_sm.pdf]

Science Advances
AAAS

Supplementary Materials for

Odd-even conductance oscillations in *meta*-cycloparaphenylenes

Xuwei Song *et al.*

Corresponding author: Jing-Tao Lü, jtlu@hust.edu.cn; Huan Cong, hcong@mail.ipc.ac.cn; Yaping Zang, zangyaping@iccas.ac.cn

Sci. Adv. **12**, eaeb8037 (2026)
DOI: 10.1126/sciadv.aeb8037

This PDF file includes:

Supplementary Text
Figs. S1 to S49
References

1. Additional Data Analysis

1.1 Conductance extraction from 2D histogram profiles

To accurately determine the conductance values at the maximum elongation of single-molecule junctions, 1D conductance histograms were constructed by integrating a 0.10 nm-wide analysis window (marked by vertical lines of the 2D conductance-displacement histogram in Fig. 4C and Supporting Information), as exemplified in Fig. 4C (*34*). The selection criterion for the vertical line is defined as 85% of the maximum plateau extension distance.

1.2 Measurement reproducibility and statistical analysis

To ensure reproducibility, five independent STM-BJ measurements were performed for each *m*[n]CPP (n = 5-10). 1D and 2D conductance histograms were constructed from the collected datasets. The conductance values were statistically analyzed following the procedures described in Supporting Information Section 1.1. Junction length distributions were extracted from the 2D histograms (*67, 68*). Statistical analysis of the five independent measurements was performed by plotting the mean conductance values and junction lengths with their corresponding standard deviation error bars (Figures S10 to S32). The consistently small error bars observed in both the conductance and length distributions provide strong evidence for the high reproducibility of the measurements.

1.3 Conductance analysis of *m*[10]CPP single molecule junctions.

A general feature of CPPs molecular junctions is that larger rings naturally offer more possible Au–π contact sites during stretching. For *m*[10]CPP, this results in a broader distribution of junction geometries and more sloped conductance traces. To ensure that our analysis is not biased, we examined multiple independent datasets and obtained consistent conductance values (Figures S23 to S26). We also repeated the extraction at different extension thresholds (80%, 85%, and 88%), and the conductance associated with the fully extended configuration remains stable across all thresholds. This confirms that the value assigned to *m*[10]CPP is robust and not dependent on manual selection (Figure S24).

1.4 Equivalent-resistor representation of electron transport in *m*CPPs junctions

As shown in Figure S46, along the transport path, the phenylene units behave as lower-resistance segments (r_1), whereas the inter-unit single bonds—where torsion reduces π overlap—act as higher-resistance segments (r_2). This picture is supported by the calculated eigenchannel distributions: within each phenylene unit, the eigenchannel amplitude is

relatively uniform, while a marked reduction of amplitude appears at the inter-ring single bonds, indicating that these positions act as local bottlenecks for charge transport. This behavior is consistent with modeling the backbone as a series of alternating low- and high-resistance elements.

In the longest transport configuration, odd-numbered *m*CPPs contain fewer high-resistance inter-unit segments on average, giving a lower overall series resistance than the even-numbered analogues. Consequently,

$$R_{\mathrm{odd}} < R_{\mathrm{even}} \text{ and } G_{\mathrm{odd}} > G_{\mathrm{even}}.$$

This simple geometric–pathway picture, together with the eigenchannel analysis, explains why the odd series shows higher contact conductance.

2. Additional Experimental Figures

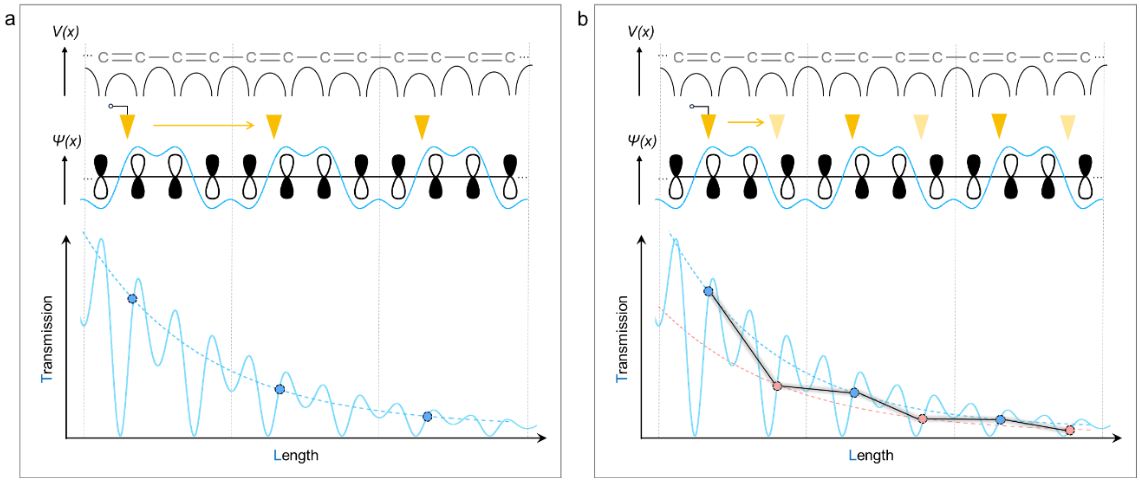

Figure S1 | The effect of molecular orbital spatial distribution on length dependent transmission. In real molecular systems, the atomic structure of the backbone introduces a non-uniform potential landscape. The resulting molecular orbitals exhibit spatially varying amplitude along the molecular axis. For example, alternating single and double C–C bonds give rise to a quasi-periodic potential and nodal patterns in the wavefunction. Consequently, the length-dependent transmission displays oscillations that reflect the spatial structure of the molecular orbital, rather than purely exponential decay. (**a**) When the sampling interval is comparable to the molecular repeat unit, the electrodes effectively probe similar orbital-amplitude regions at each length. Because the local amplitudes sampled remain nearly the same, the transmission follows a single smooth exponential decay. (**b**) When the sampling interval becomes finer than the spatial modulation of the orbital amplitude, different contact positions probe distinct amplitude levels within the modulation pattern. Each set of sampled positions gives rise to its own exponential decay trend, producing two separate decay envelopes (blue and red dashed lines). The alternation between these branches as molecular length increases gives rise to the observed odd–even conductance oscillations.

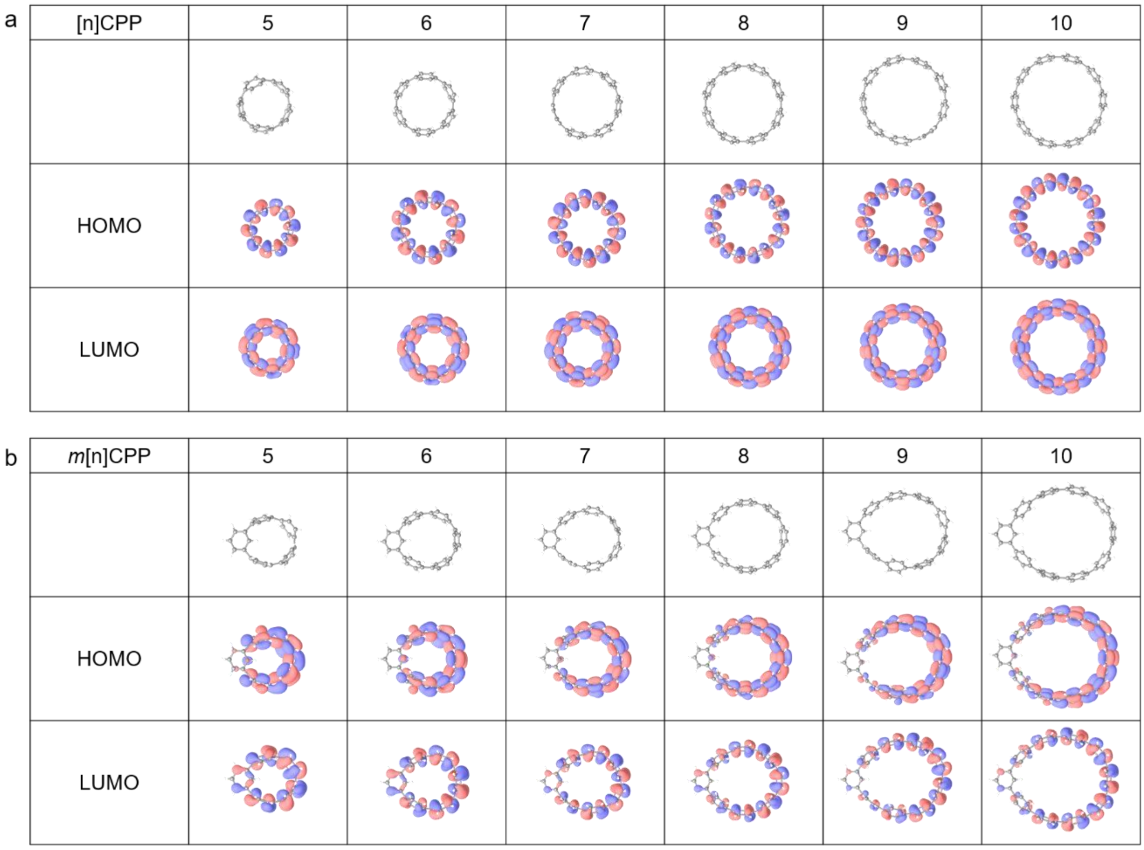

Figure S2 | Molecular geometries and orbitals of [n]CPPs and *m*[n]CPPs (n=5-10). (**a**) Molecular geometries and orbitals of [n]CPPs. (**b**) Molecular geometries and orbitals of *m*[n]CPPs. The introduction of a *meta*-substituted phenylene disrupts the delocalization in CPP, but does not change the nodal topology of its frontier orbitals. Both CPPs and *m*CPPs display frontier orbitals with similarly resolved nodal topologies.

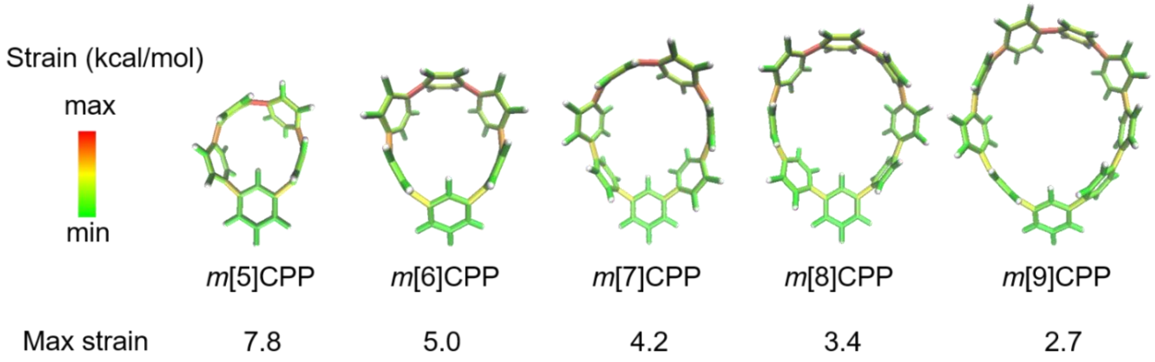

Figure S3 | Strain energy distribution in *m*[n]CPPs (n =5-9). The calculated strain energy distribution in *m*[n]CPPs is visualized with individual bonds color-coded according to strain intensity: high (red), medium (yellow), and low (green). The numerical values at the bottom indicate the maximum bond strain energy for each *m*[n]CPP. All calculations were performed

at the DFT/B3LYP level using Gaussian 16, with strain analysis and visualization conducted via StrainViz.

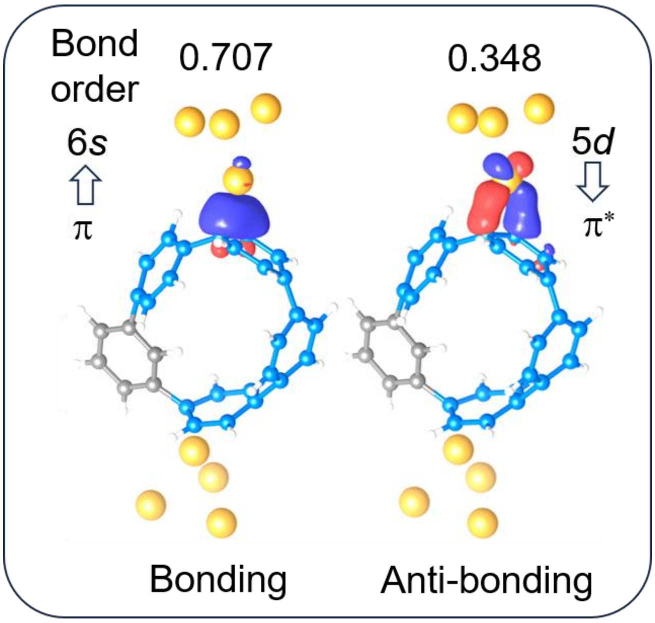

Figure S4 | NAdO analysis for the bond between *m*[5]CPP and Au clusters calculated using the Multiwfn package. The results reveal a direct η^2-type Au–π coordination between the gold electrode and individual C=C bonds within the phenylene rings. Both electron donation and back-donation processes contribute to the binding: π orbitals of *m*[5]CPP donate electron density to the Au 6*s* orbitals, while back-donation from Au 5*d* orbitals into the π^* orbitals further stabilizes the interaction.

	m[5]CPP+Au	*m*[5]CPP
HOMO	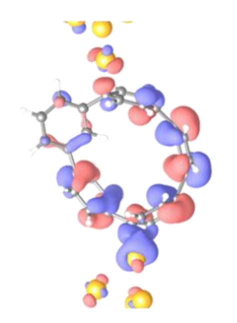	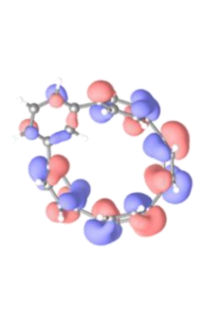
LUMO	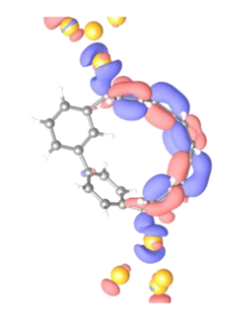	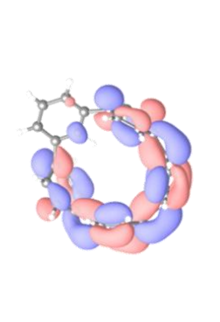

Figure S5 | Orbitals of *m*[5]CPP and *m*[5]CPP junction. HOMO and LUMO of *m*[5]CPP and scattering state of *m*[5]CPP junction. Comparing the molecular orbitals before and after the attachment of gold electrodes reveals that the orbital symmetry remains unchanged.

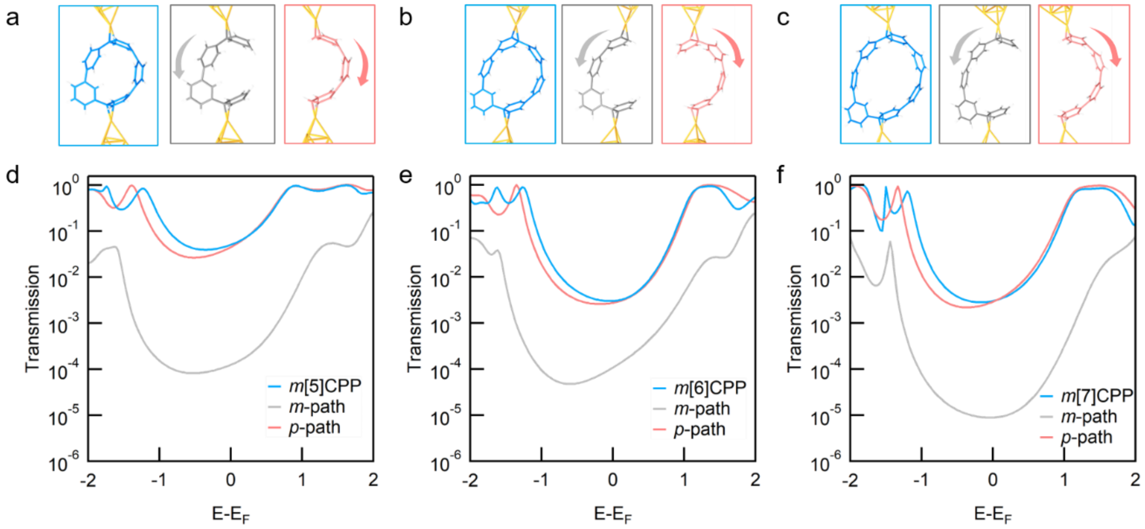

Figure S6 | DFT calculated electron transmission spectra for *m*[n]CPPs (n=5-7). (a-c) Junction geometries used for computing the transmission spectra of the *m*[n]CPPs (n=5-7, blue), *meta*-connected oligophenylene (gray) and *para*-connected oligophenylene (red) brunches. (**d-f**) The total molecular transmission (blue) is dominated almost entirely by the *para*-connected oligophenylene backbone (red), while the *meta*-connected side (gray) contributes minimally. A *meta*-connected benzene unit was incorporated into the ring to break molecular symmetry. This demonstrates that the incorporation of a *meta*-connected benzene unit effectively suppresses one transport pathway while stabilizing the overall junction geometry, resulting in a single, well-defined conduction channel through a coherent π-conjugated backbone.

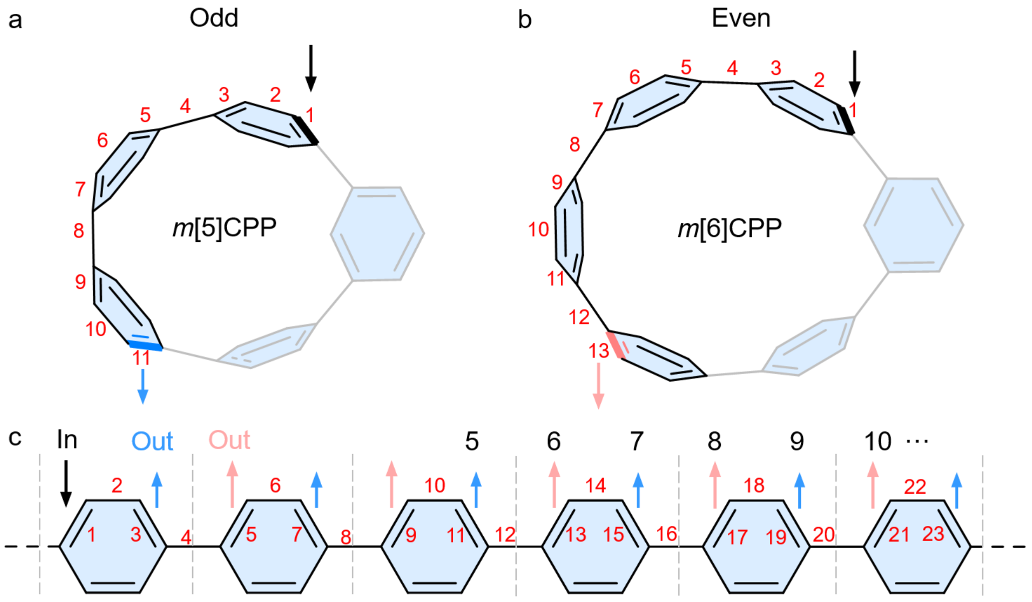

Figure S7 | Schematic illustration of how molecular geometry dictates the transport pathway in *m*[n]CPPs. (**a**) For odd-membered rings (e.g., *m*[5]CPP), electron injection (black arrow) leads to an output through a C=C bond located on the lower side of a phenylene ring (blue arrow). (**b**) In even-membered rings (e.g., *m*[6]CPP), the output shifts to the upper-side C=C bond (red arrow), reflecting a parity-induced alternation in spatial arrangement. (**c**) Linearized representations of the *m*[n]CPP structures illustrate how this odd–even variation leads to a systematic shift in the output position along the oligophenylene backbone. Arrows indicate electrode contacts (black: input; blue/red: output). This structural analysis reveals that the geometric parity of the nanohoop directly determines the through-bond conjugation route—shifting the dominant transport pathway between alternating C=C bonds.

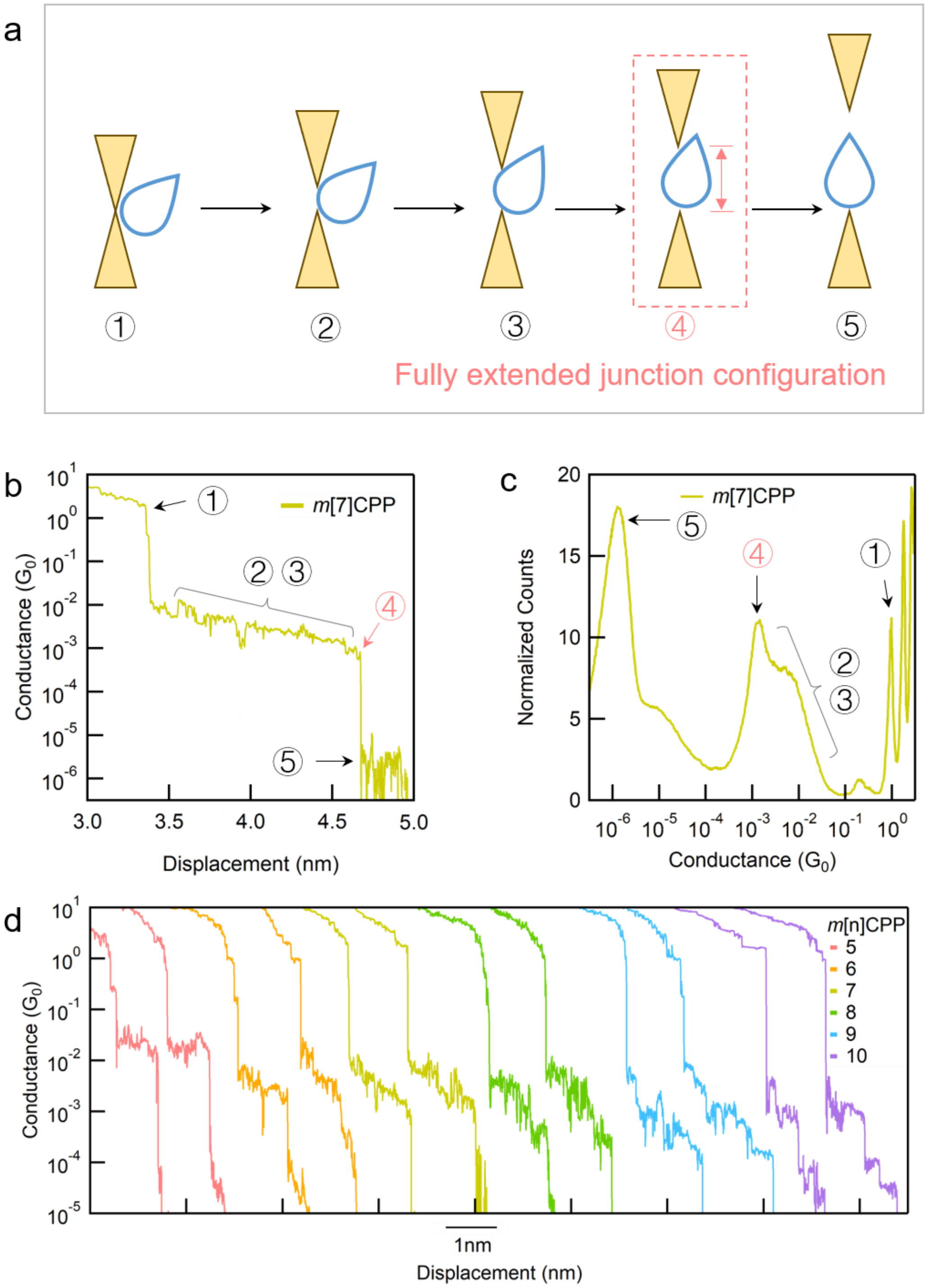

Figure S8 | STM-BJ measurement results of *m*[5]CPP-*m*[10]CPP. (a) Schematic illustration of junction evolution during the break-junction process as the STM tip is gradually retracted. Configuration ①: Initial rupture of the Au–Au contact. Configurations ② and ③: Gold electrodes slide along the carbon backbone, forming transient molecule–electrode junctions at various sites. Configuration ④: Fully extended junction, in which the electrodes anchor at opposite ends of the molecule, resulting in maximal separation. Configuration ⑤: Final rupture of the molecular junction. (**b**) A representative conductance–displacement trace

for *m*[7]CPP, showing sloped plateaus corresponding to transitions among these configurations. (**c**) 1D conductance histogram for *m*[7]CPP, constructed from thousands of individual traces. (**d**) Representative conductance traces for *m*[5]CPP to *m*[10]CPP, measured at an applied tip bias of 100 mV. As the ring size increases, the conductance plateaus become increasingly sloped, reflecting a larger number of accessible contact geometries along the extended carbon backbone. Based on this behavior and the structural analysis above, the fully extended configuration (④) is identified as the representative geometry for extracting molecular conductance values in subsequent analysis. To ensure consistency, the histogram integration window (0.10 nm wide, as shown in Fig. 4B) is centered around the displacement region corresponding to configuration ④.

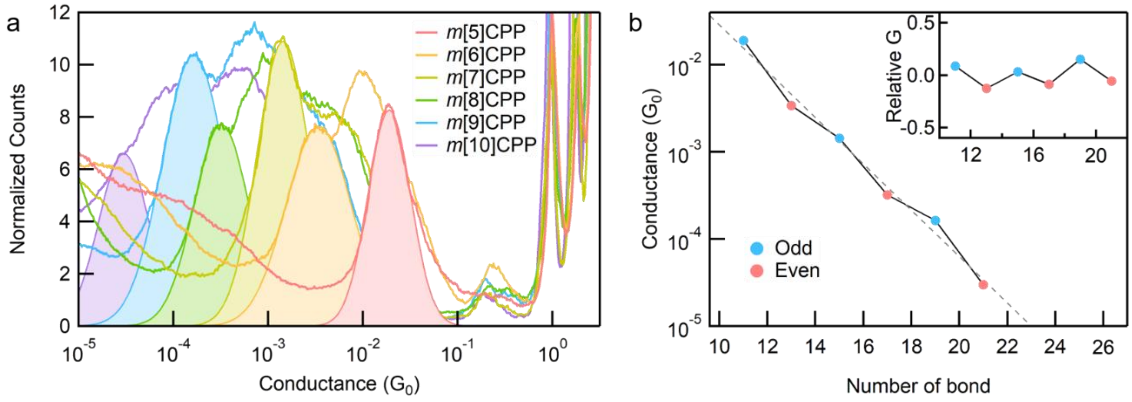

Figure S9 | Single-molecule conductance of *m*[5]CPP-*m*[10]CPP. (**a**) 1D conductance histograms for *m*[5]CPP to *m*[10]CPP. (**b**) Single-molecule conductance of *m*[n]CPPs (n = 5-10) plotted against the number of bonds obtained from molecular geometries (see Figure S7). The overall trend follows an exponential decay with increasing junction length (gray dashed line), while a distinct odd-even oscillation is superimposed. Blue and red dots denote odd- and even-membered *m*[n]CPPs, respectively, consistent with the color coding used in the main text. Inset: Relative conductance values defined as G_{rel}=G_{exp}-G_{fit}, where G_{fit} is the baseline exponential decay. The nearly constant amplitude of the oscillation across molecular lengths demonstrates the structural robustness of the parity-induced conductance modulation.

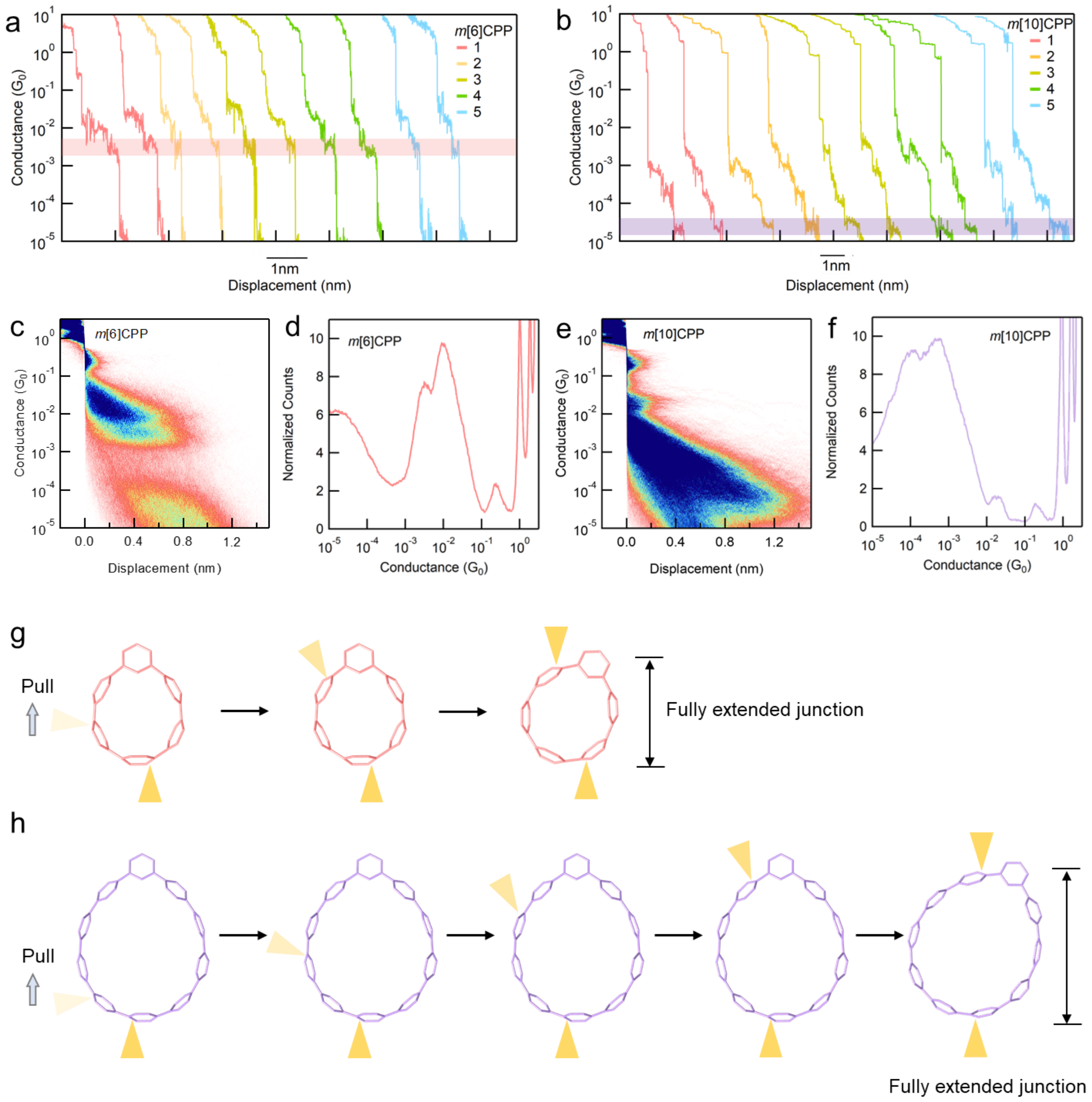

Figure S10 | Conductance characteristics and junction evolution of *m*[6]CPP and *m*[10]CPP single-molecule junctions. (**a, b**) Representative individual conductance-displacement traces of *m*[6]CPP and *m*[10]CPP. (**c-f**) 2D and 1D conductance histograms of *m*[6]CPP and *m*[10]CPP. (**g, h**) Schematic illustrations of the evolution of the molecular configuration during the stretching process, showing the final fully extended configuration with the longest Au–Au distance.

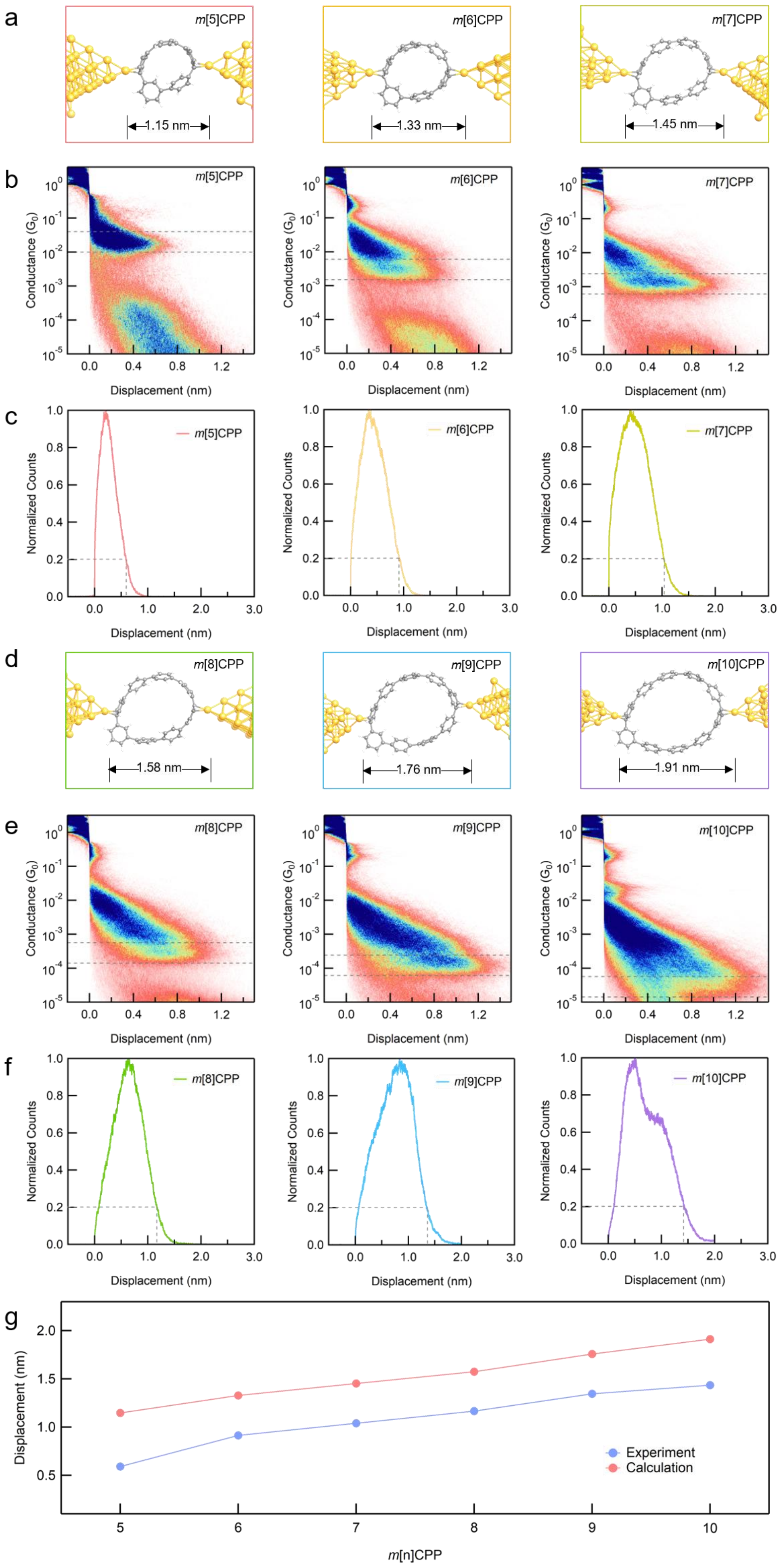
a
m[5]CPP
1.15 nm
m[6]CPP
1.33 nm
m[7]CPP
1.45 nm
b
Conductance (G0)
Displacement (nm)
c
Normalized Counts
Displacement (nm)
d
m[8]CPP
1.58 nm
m[9]CPP
1.76 nm
m[10]CPP
1.91 nm
e
f
g
Displacement (nm)
Experiment
Calculation
m[n]CPP

Figure S11 | Junction length analysis of *m*[n]CPPs (n = 5–10). (**a, d**) DFT-optimized structures of *m*[n]CPP (n = 5-10) single-molecule junctions with gold electrodes. The calculated Au-Au distances are 1.15 nm (*m*[5]CPP), 1.33 nm (*m*[6]CPP), 1.45 nm (*m*[7]CPP), 1.58 nm (*m*[8]CPP), 1.76 nm (*m*[9]CPP), and 1.91 nm (*m*[10]CPP). (**b, e**) 2D histograms for *m*[5]CPP to *m*[10]CPP. The horizontal dashed lines indicate the integration windows used to profile junction lengths. (**c, f**) Normalized displacement distributions derived from the 2D histograms. The 80th-percentile displacement values (marked by dashed lines) are used to represent the experimental junction lengths for each *m*[n]CPP. (**g**) Comparison between the experimentally extracted and DFT-calculated Au-Au junction lengths. The experimental values are systematically shorter due to a typical ~0.5 nm snap-back distance of gold electrodes following contact rupture (*69*). After including this correction, the experimental and calculated lengths show excellent agreement, supporting the formation of fully extended molecular junctions in STM-BJ measurements.

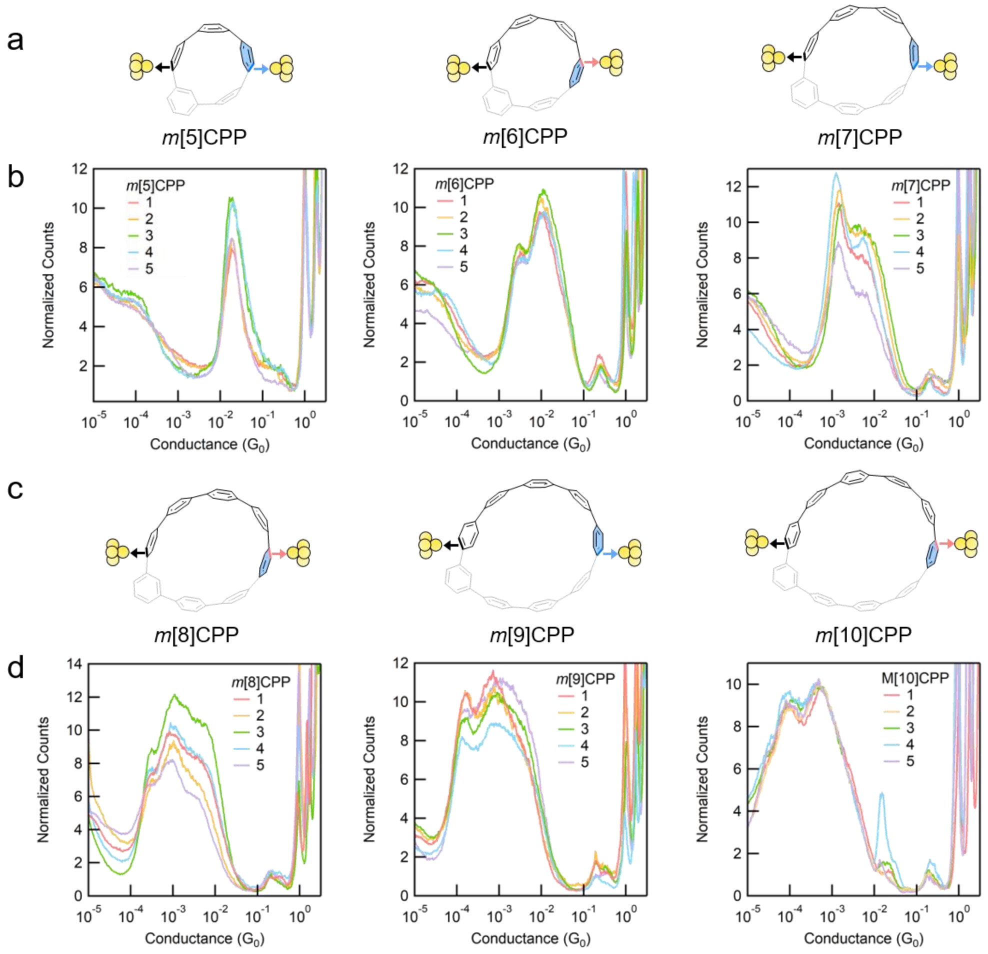

Figure S12 | Reproducibility of STM-BJ measurements for *m*[5]CPP-*m*[10]CPP. (**a, c**) Schematic illustrations of single-molecule junction configurations for m[n]CPPs (n =5–10). (**b, d**) 1D conductance histograms for *m*[5]CPP-*m*[10]CPP, each obtained from five independent

STM-BJ experimental runs (labeled 1–5). Each histogram is compiled from over 5,000 individual conductance-displacement traces without data selection. The high consistency among repeated measurements confirms the robustness and reproducibility of the experimental procedure and analysis.

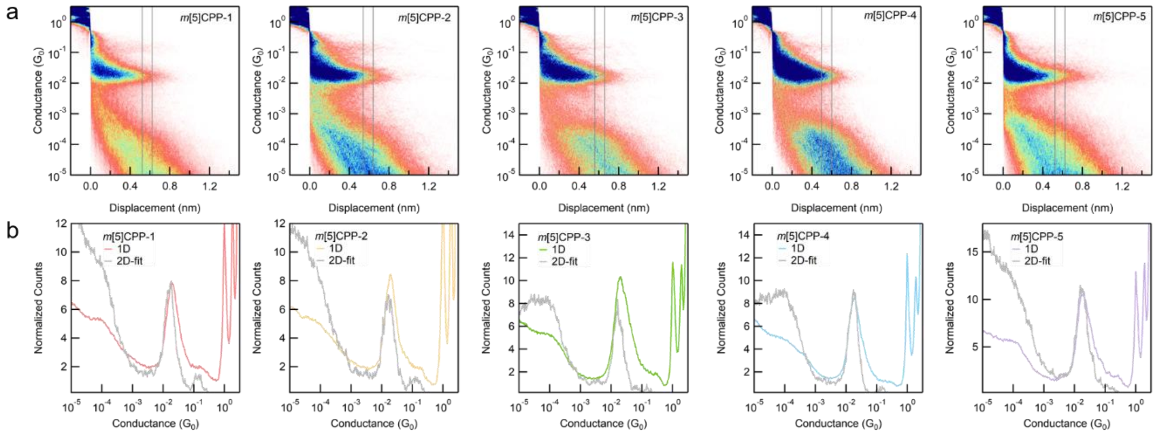

Figure S13 | 1D and 2D conductance histograms from five independent STM-BJ measurements of *m*[5]CPP. (**a**) 2D histograms from five separate measurements of *m*[5]CPP. Vertical dashed lines indicate the displacement window used for extracting conductance values corresponding to the fully extended molecular configuration. (**b**) Comparison between the full 1D conductance histograms (colored curves) and the conductance profiles (grey curves) obtained by vertical integration within the selected displacement window in (**a**). The strong agreement across all five datasets confirms the reproducibility and statistical robustness of the extracted molecular conductance.

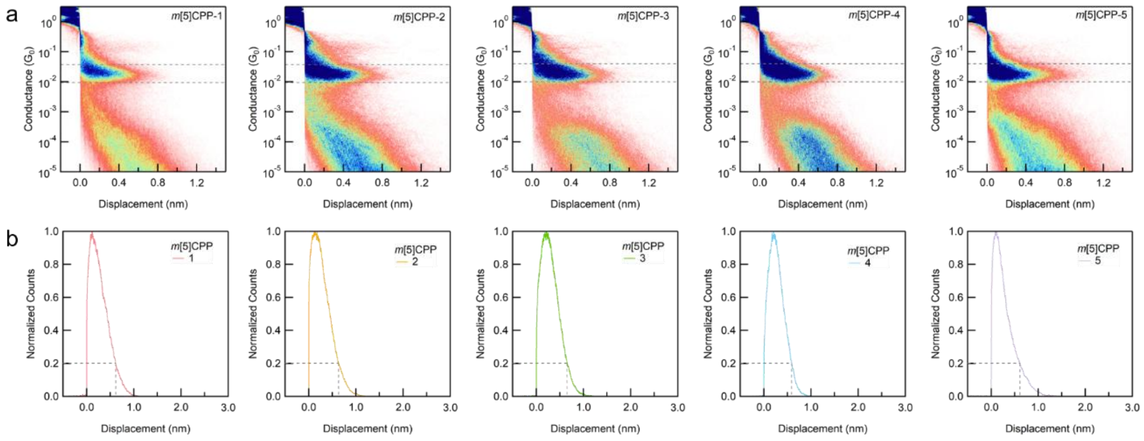

Figure S14 | Junction length analysis of *m*[5]CPP. (**a**) 2D histograms from five independent measurements of *m*[5]CPP. The horizontal dashed lines indicate the integration windows used to profile junction lengths. (**b**) Normalized displacement distributions derived from the 2D

histograms. The 80th-percentile displacement values (marked by dashed lines) are used to represent the experimental junction lengths for each *m*[5]CPP.

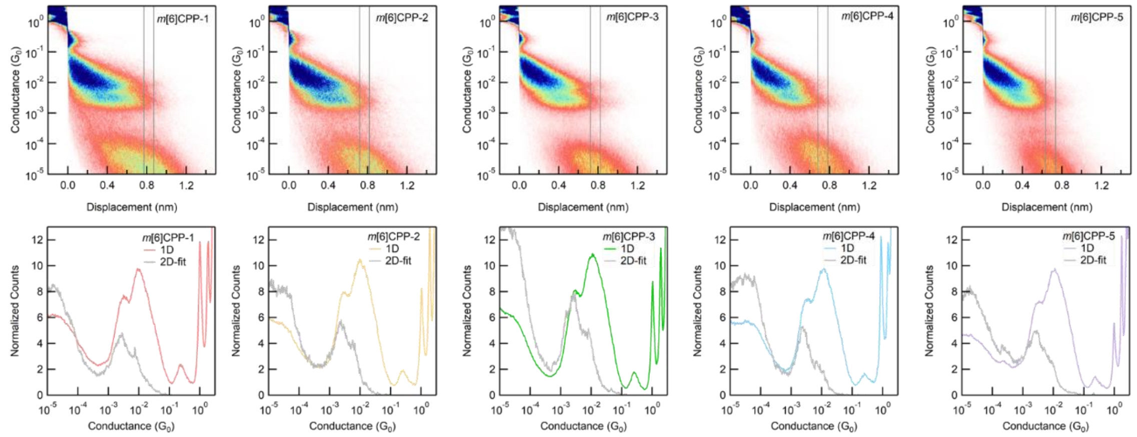

Figure S15 | 1D and 2D conductance histograms from five independent STM-BJ measurements of *m*[6]CPP. (**a**) 2D histograms from five separate measurements of *m*[6]CPP. Vertical dashed lines indicate the displacement window used for extracting conductance values corresponding to the fully extended molecular configuration. (**b**) Comparison between the full 1D conductance histograms (colored curves) and the conductance profiles (grey curves) obtained by vertical integration within the selected displacement window in (**a**). The strong agreement across all five datasets confirms the reproducibility and statistical robustness of the extracted molecular conductance.

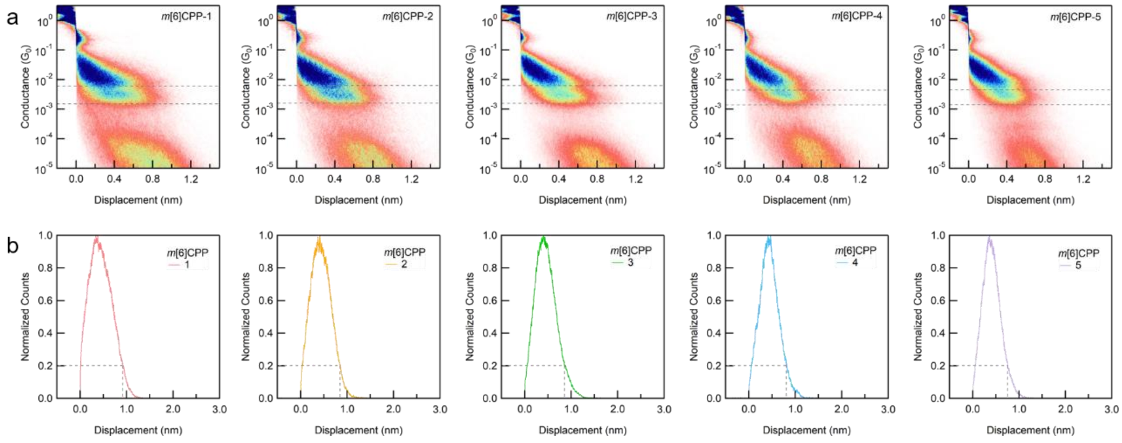

Figure S16 | Junction length analysis of *m*[6]CPP. (**a**) 2D histograms from five independent measurements of *m*[6]CPP. The horizontal dashed lines indicate the integration windows used to profile junction lengths. (**b**) Normalized displacement distributions derived from the 2D histograms. The 80th-percentile displacement values (marked by dashed lines) are used to represent the experimental junction lengths for each *m*[6]CPP.

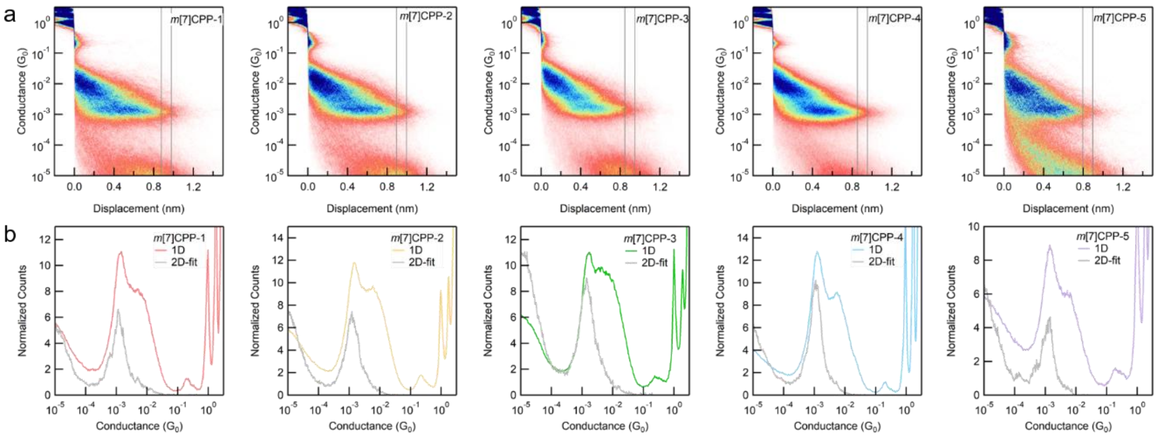

Figure S17 | 1D and 2D conductance histograms from five independent STM-BJ measurements of *m*[7]CPP. (**a**) 2D histograms from five separate measurements of *m*[7]CPP. Vertical dashed lines indicate the displacement window used for extracting conductance values corresponding to the fully extended molecular configuration. (**b**) Comparison between the full 1D conductance histograms (colored curves) and the conductance profiles (grey curves) obtained by vertical integration within the selected displacement window in (**a**). The strong agreement across all five datasets confirms the reproducibility and statistical robustness of the extracted molecular conductance.

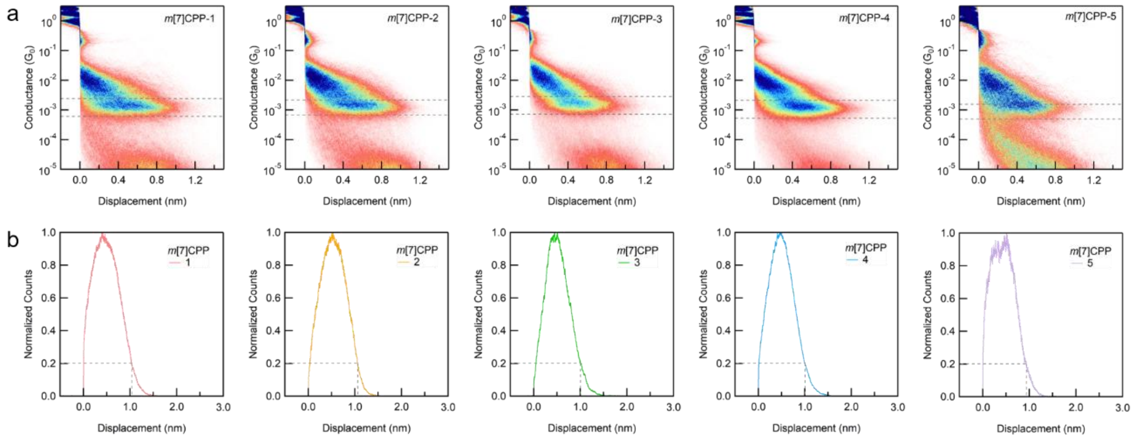

Figure S18 | Junction length analysis of *m*[7]CPP. (**a**) 2D histograms from five independent measurements of *m*[7]CPP. The horizontal dashed lines indicate the integration windows used to profile junction lengths. (**b**) Normalized displacement distributions derived from the 2D histograms. The 80th-percentile displacement values (marked by dashed lines) are used to represent the experimental junction lengths for each *m*[7]CPP.

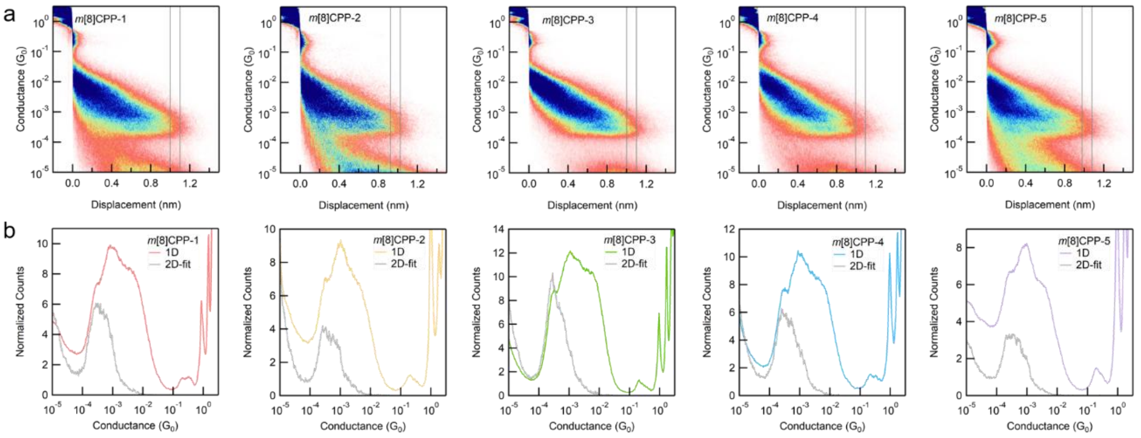

Figure S19 | 1D and 2D conductance histograms from five independent STM-BJ measurements of *m*[8]CPP. (**a**) 2D histograms from five separate measurements of *m*[8]CPP. Vertical dashed lines indicate the displacement window used for extracting conductance values corresponding to the fully extended molecular configuration. (**b**) Comparison between the full 1D conductance histograms (colored curves) and the conductance profiles (grey curves) obtained by vertical integration within the selected displacement window in (**a**). The strong agreement across all five datasets confirms the reproducibility and statistical robustness of the extracted molecular conductance.

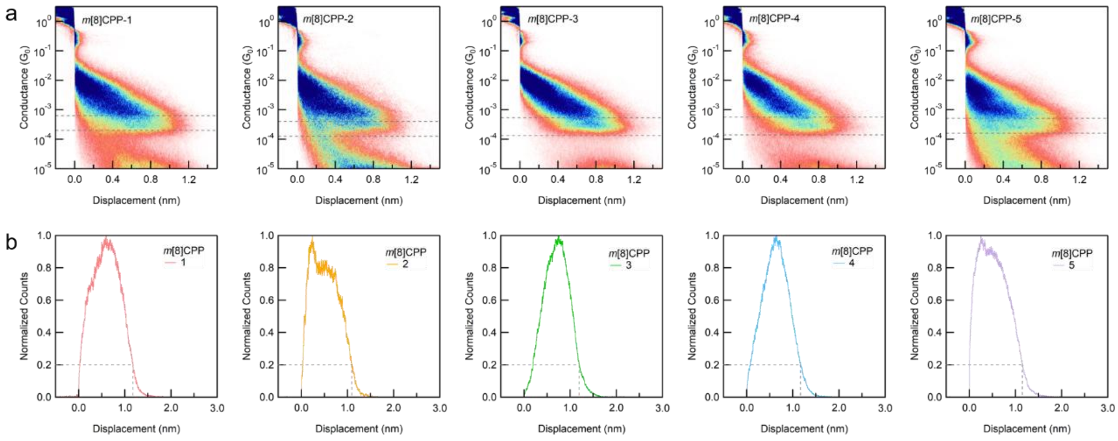

Figure S20 | Junction length analysis of *m*[8]CPP. (**a**) 2D histograms from five independent measurements of *m*[8]CPP. The horizontal dashed lines indicate the integration windows used to profile junction lengths. (**b**) Normalized displacement distributions derived from the 2D histograms. The 80th-percentile displacement values (marked by dashed lines) are used to represent the experimental junction lengths for each *m*[8]CPP.

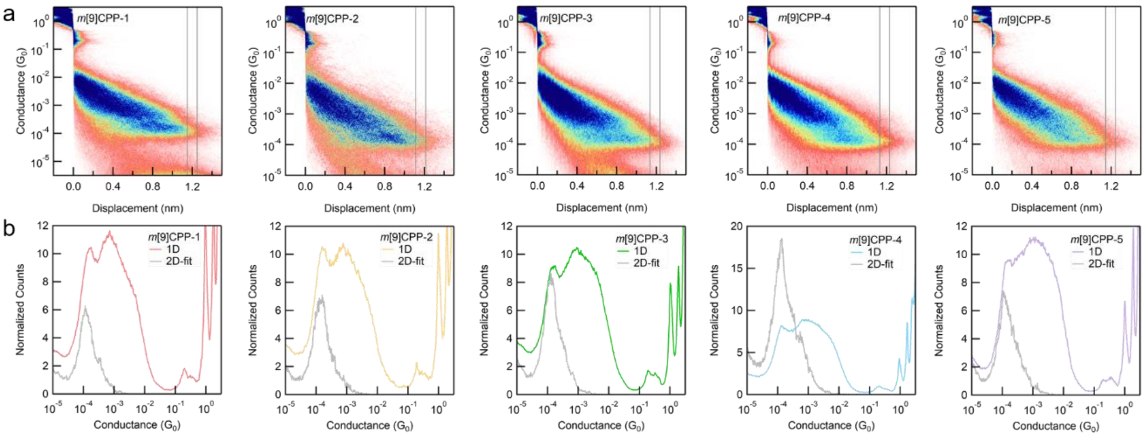

Figure S21 | 1D and 2D conductance histograms from five independent STM-BJ measurements of *m*[9]CPP. (**a**) 2D histograms from five separate measurements of *m*[9]CPP. Vertical dashed lines indicate the displacement window used for extracting conductance values corresponding to the fully extended molecular configuration. (**b**) Comparison between the full 1D conductance histograms (colored curves) and the conductance profiles (grey curves) obtained by vertical integration within the selected displacement window in (**a**). The strong agreement across all five datasets confirms the reproducibility and statistical robustness of the extracted molecular conductance.

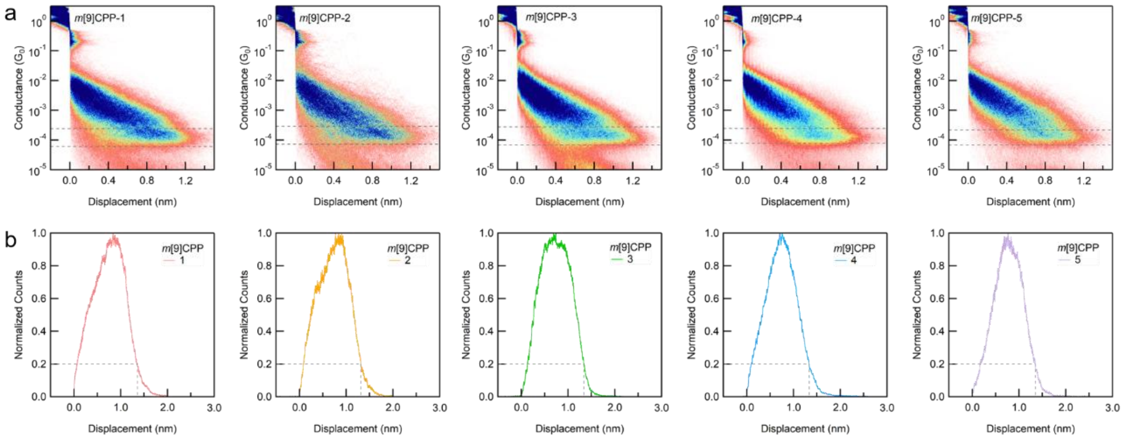

Figure S22 | Junction length analysis of *m*[9]CPP. (**a**) 2D histograms from five independent measurements of *m*[9]CPP. The horizontal dashed lines indicate the integration windows used to profile junction lengths. (**b**) Normalized displacement distributions derived from the 2D histograms. The 80th-percentile displacement values (marked by dashed lines) are used to represent the experimental junction lengths for each *m*[9]CPP.

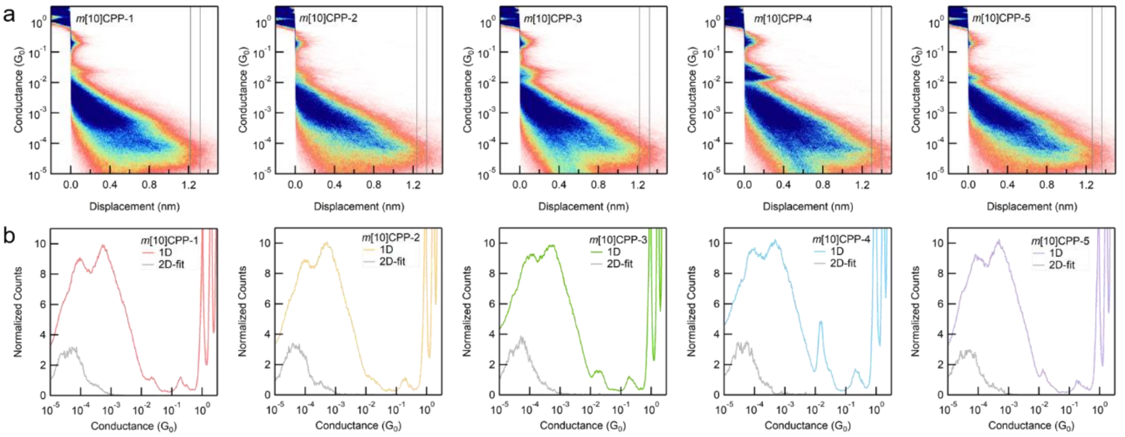

Figure S23 | 1D and 2D conductance histograms from five independent STM-BJ measurements of *m*[10]CPP. (**a**) 2D histograms from five separate measurements of *m*[10]CPP. Vertical dashed lines indicate the displacement window used for extracting conductance values corresponding to the fully extended molecular configuration. (**b**) Comparison between the full 1D conductance histograms (colored curves) and the conductance profiles (grey curves) obtained by vertical integration within the selected displacement window in (**a**). The strong agreement across all five datasets confirms the reproducibility and statistical robustness of the extracted molecular conductance.

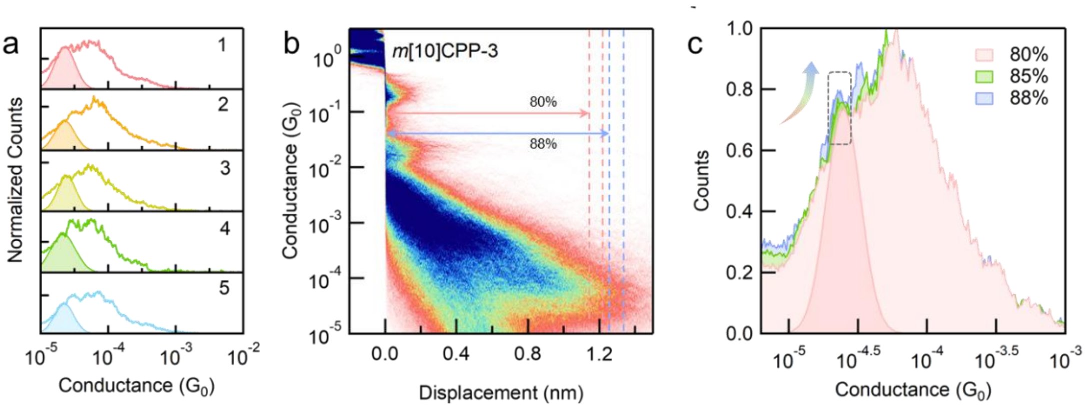

Figure S24 | Conductance characteristics of *m*[10]CPP single molecule junctions. (**a**) Overlaid 1D conductance histograms from five independent STM-BJ measurements for *m*[10]CPP. The histograms were extracted using the displacement window corresponding to the fully extended junction configuration, as defined in the 2D histograms. (**b**) 2D conductance-displacement histogram for *m*[10]CPP. The vertical lines at the end of the molecular feature indicate the window for determining the conductance profiles presented in (c), defined at 80%, 85% and 88% of the maximum plateau extension distance. (**c**) Comparison of the conductance profiles at different extension thresholds, showing the robust and consistent conductance feature at the fully extended configuration.

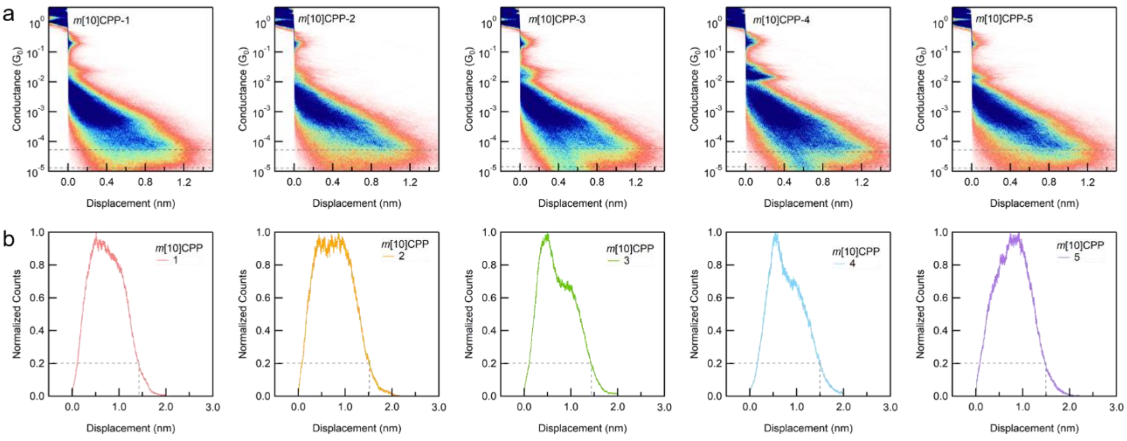

Figure S25 | Junction length analysis of *m*[10]CPP. (**a**) 2D histograms from five independent measurements of *m*[10]CPP. The horizontal dashed lines indicate the integration windows used to profile junction lengths. (**b**) Normalized displacement distributions derived from the 2D histograms. The 80th-percentile displacement values (marked by dashed lines) are used to represent the experimental junction lengths for each *m*[10]CPP.

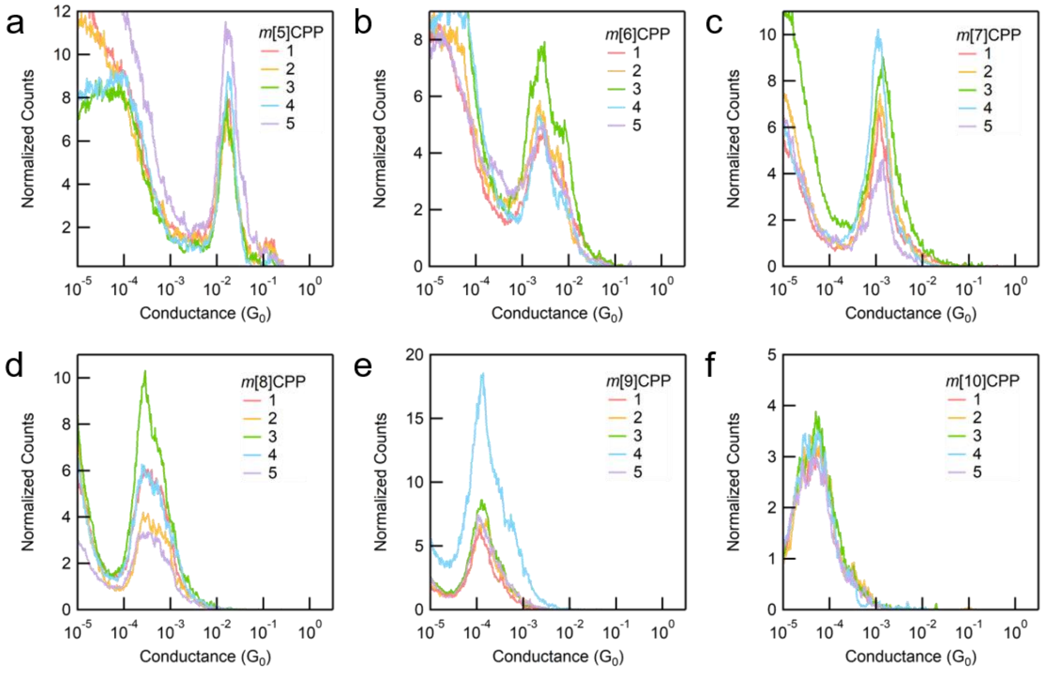

Figure S26 | Reproducibility of extracted single-molecule conductance for *m*[5]CPP to *m*[10]CPP. Overlaid 1D conductance histograms from five independent STM-BJ measurements for each *m*[n]CPP (n = 5-10). The histograms were extracted using the displacement window corresponding to the fully extended junction configuration, as defined in the 2D histograms shown in previous figures. The strong consistency across all datasets confirms the reproducibility and statistical reliability of the extracted single-molecule

conductance values. These profiles serve as the basis for Gaussian fitting in the conductance analysis shown in Figure S27.

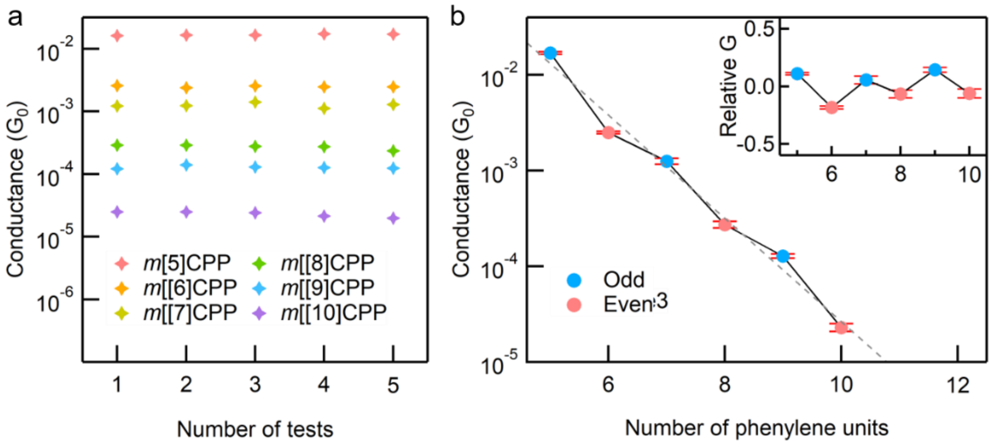

Figure S27 | Statistical analysis of single-molecule conductance for *m*[5]CPP to *m*[10]CPP. (**a**) Conductance values extracted by Gaussian fitting of each individual dataset from the five independent STM-BJ measurements shown in Figure S26. (**b**) Mean conductance values with standard deviation error bars for each *m*[n]CPP, plotted as a function of ring size. Blue and red symbols represent odd- and even-membered *m*[n]CPPs, respectively. The results exhibit a clear exponential decay trend with increasing ring size, accompanied by a pronounced odd-even conductance oscillation. Inset: Relative conductance values defined as G_{rel}=G_{exp}-G_{fit}, where G_{fit} is the baseline exponential decay. The nearly constant amplitude of the oscillation across number of phenylene units demonstrates the structural robustness of the parity-induced conductance modulation.

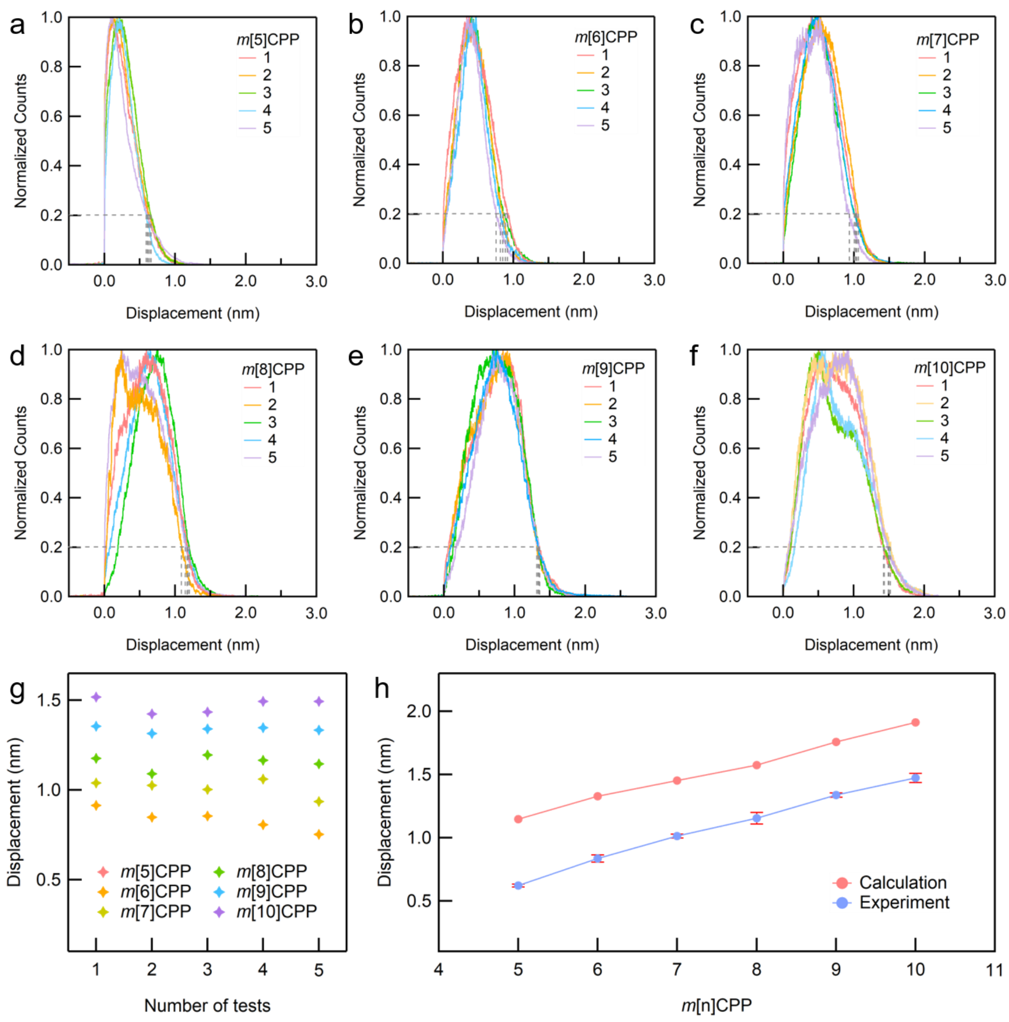

Figure S28 | Reproducibility of extracted single-molecule junction length for *m*[5]CPP to *m*[10]CPP. (**a-f**) Overlaid normalized displacement distributions from five independent STM-BJ measurements for each *m*[n]CPP (n = 5-10). The 80th-percentile displacement values (marked by dashed lines) are used to represent the experimental junction lengths for each *m*[n]CPP. The strong consistency across all datasets confirms the reproducibility and statistical reliability of the extracted single-molecule junction length values. (**g**) Single-molecule junction length values extracted of each individual dataset from the five independent STM-BJ measurements shown in (**a-f**). (**h**) Comparison between the experimentally extracted junction lengths with standard deviation error bars and DFT-calculated Au-Au junction lengths. The experimental values are systematically shorter due to a typical ~0.5 nm snap-back distance of gold electrodes following contact rupture. After including this correction, the experimental and calculated lengths show excellent agreement, supporting the formation of fully extended molecular junctions in STM-BJ measurements. The high consistency among repeated measurements confirms the robustness and reproducibility of the experimental procedure and analysis.

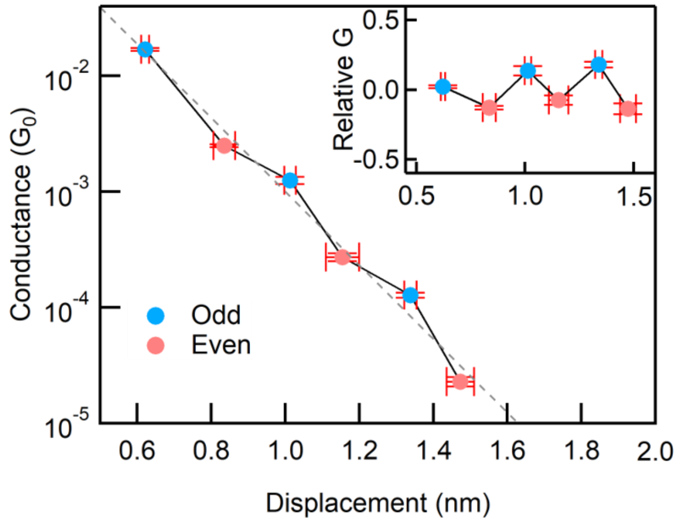

Figure S29 | Odd–even conductance oscillations as a function of the experimentally measured junction length. Mean conductance values with standard deviation error bars for each *m*[n]CPP, plotted as a function of the experimentally measured junction mean length values with standard deviation error bars. Blue and red symbols represent odd- and even-membered *m*[n]CPPs, respectively. The results exhibit a clear exponential decay trend with increasing junction lengths, accompanied by a pronounced odd–even conductance oscillation. Inset: Relative conductance values defined as $G_{rel}=G_{exp}-G_{fit}$, where G_{fit} is the baseline exponential decay. The nearly constant amplitude of the oscillation across molecular lengths demonstrates the structural robustness of the parity-induced conductance modulation.

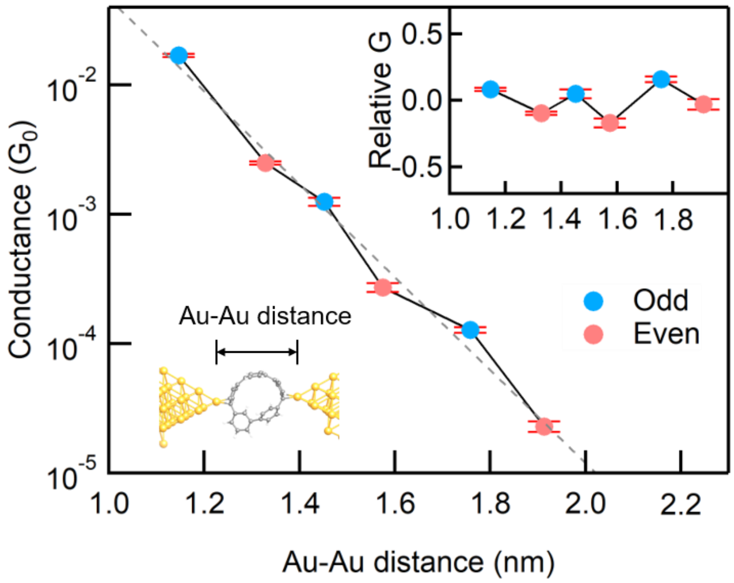

Figure S30 | Odd-even conductance oscillations as a function of the DFT-calculated junction length (Au–Au distance). Single-molecule conductance of *m*[n]CPPs (n = 5-10) plotted against the Au-Au distance obtained from DFT-optimized junction geometries (see

Figure S11). The overall trend follows an exponential decay with increasing junction length (gray dashed line), while a distinct odd-even oscillation is superimposed. Blue and red dots denote odd- and even-membered CPPs, respectively, consistent with the color coding used in the main text. Inset: Relative conductance values defined as G_{rel}=G_{exp}-G_{fit}, where G_{fit} is the baseline exponential decay. The nearly constant amplitude of the oscillation across molecular lengths demonstrates the structural robustness of the parity-induced conductance modulation.

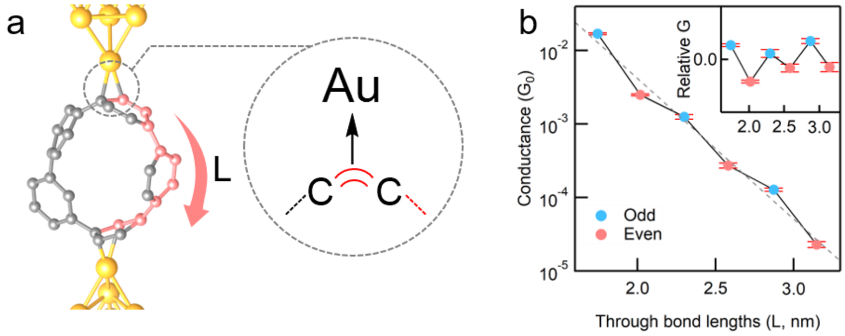

Figure S31 | Odd–even conductance oscillations as a function of the sum of through-bond lengths within transmission pathways. (**a**) Schematic illustrations of *m*[n]CPP junction configurations (n = 5-10). Gray regions denote non-dominant transmission segments and red highlights the electron-conducting bonds. Zoom in: Binding site of the C=C double bond to the gold electrode. (**b**) Mean conductance values with standard deviation error bars for each *m*[n]CPP, plotted as a function of the sum of through-bond length values. Blue and red symbols represent odd- and even-membered *m*[n]CPPs, respectively. The results exhibit a clear exponential decay trend with increasing the sum of through-bond lengths, accompanied by a pronounced odd–even conductance oscillation. Inset: Relative conductance values defined as G_{rel}=G_{exp}-G_{fit}, where G_{fit} is the baseline exponential decay. The nearly constant amplitude of the oscillation across molecular lengths demonstrates the structural robustness of the parity-induced conductance modulation.

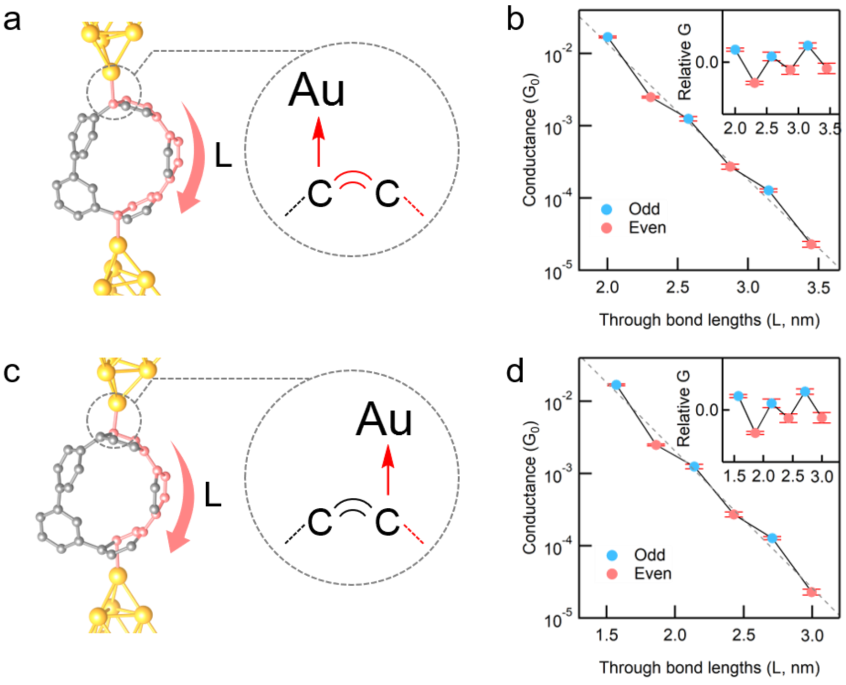

Figure S32 | Odd–even conductance oscillations as a function of the sum of through-bond lengths within transmission pathways. (**a, c**) Schematic illustrations of *m*[n]CPP junction configurations (n = 5-10). Gray regions denote non-dominant transmission segments and red highlights the electron-conducting bonds. Zoom in: binding site of the atom C to the gold electrode (The length of bonds through which the electrons flow is longest for a and the length of bonds through which the electrons flow is shortest for (c). (**b, d**) Mean conductance values with standard deviation error bars for each *m*[n]CPP, plotted as a function of the sum of through-bond length values. Blue and red symbols represent odd- and even-membered *m*[n]CPPs, respectively. The results exhibit a clear exponential decay trend with increasing the sum of through-bond lengths, accompanied by a pronounced odd–even conductance oscillation. Inset: Relative conductance values defined as G_{rel}=G_{exp}-G_{fit}, where G_{fit} is the baseline exponential decay. To eliminate potential artifacts arising from overly idealized junction geometries, we analyzed two representative limiting configurations where the electrodes are bound to C atoms on either side of the C=C bond. The persistence of conductance oscillations across these models confirms their robustness, indicating that such behavior does not depend on precise atomic alignment.

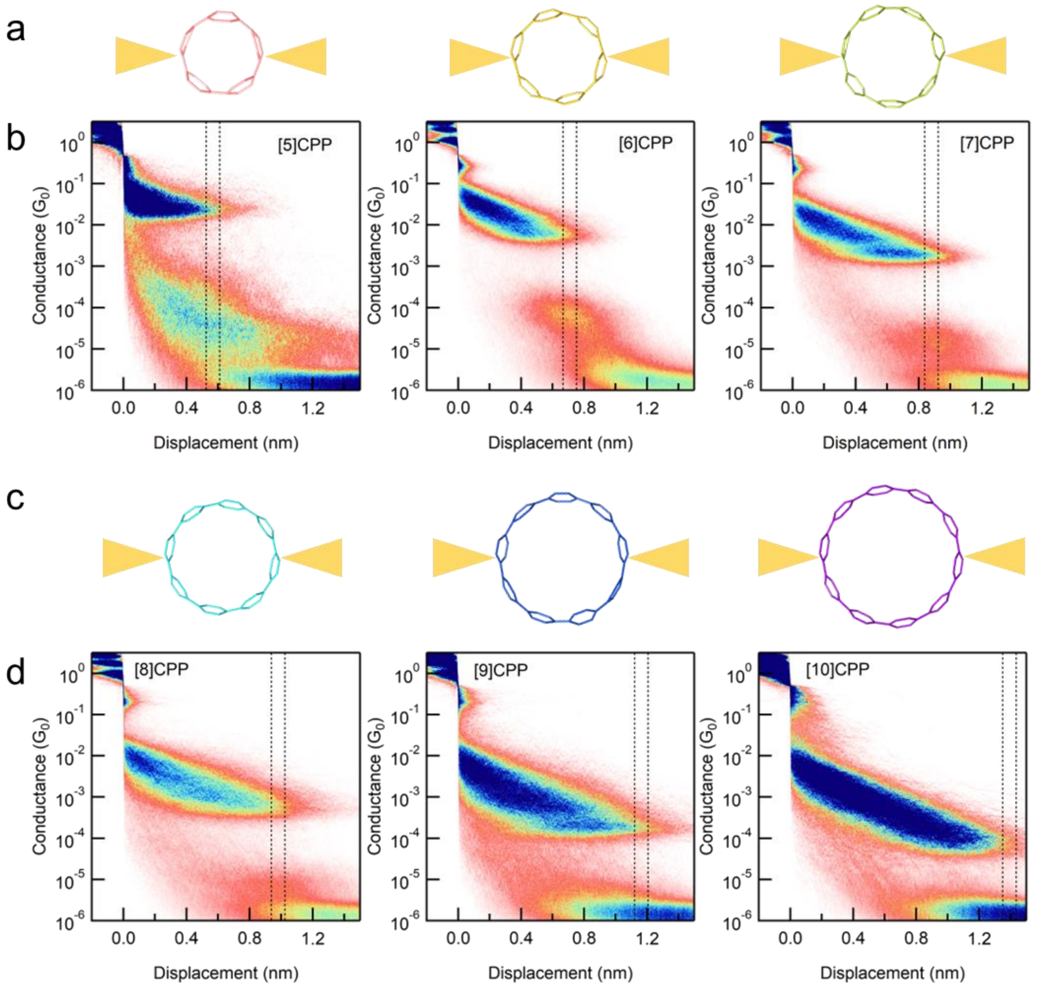

Figure S33 | 2D conductance histograms of [n]CPPs (n = 5–10). (**a, c**) Schematic illustrations of single-molecule junctions for [n]CPPs (n = 5–10). (**b, d**) Experimental 2D conductance-displacement histograms for [n]CPPs with different sizes. Vertical dashed lines indicate the displacement window used for extracting conductance values corresponding to the fully extended molecular junction configuration.

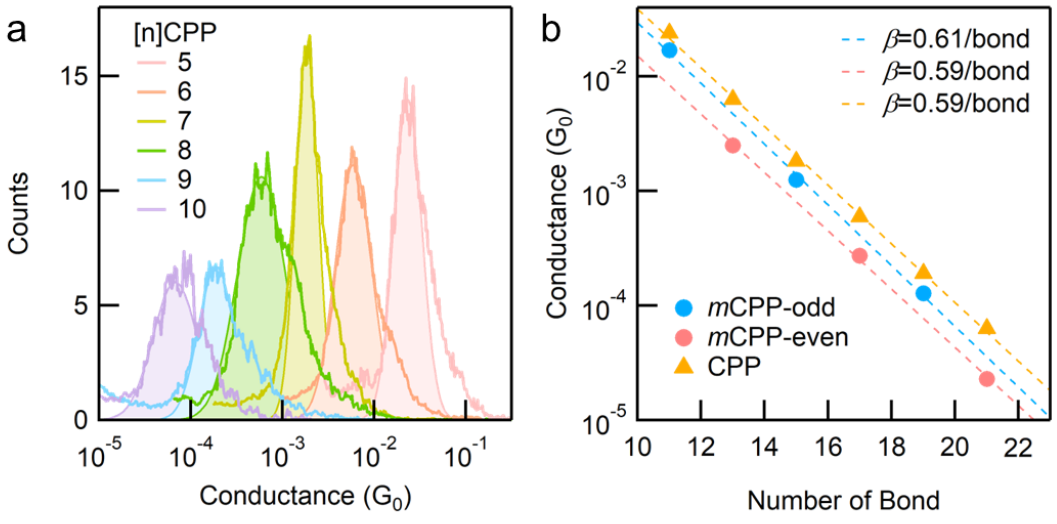

Figure S34 | Conductance characteristics of [n]CPPs (n = 5–10). (**a**) 1D conductance distribution of [n]CPPs molecules determined from the profiles in the 2D histograms. (**b**) Bond-dependent conductance plots for CPPs and *m*CPPs. While CPPs follow a single exponential decay with no detectable odd–even modulation, the *m*CPPs series clearly separates into odd and even branches. The distinct branching in *m*CPPs, contrasted with the smooth trend in CPPs, highlights the pronounced odd–even effect enabled by *meta*-substitution.

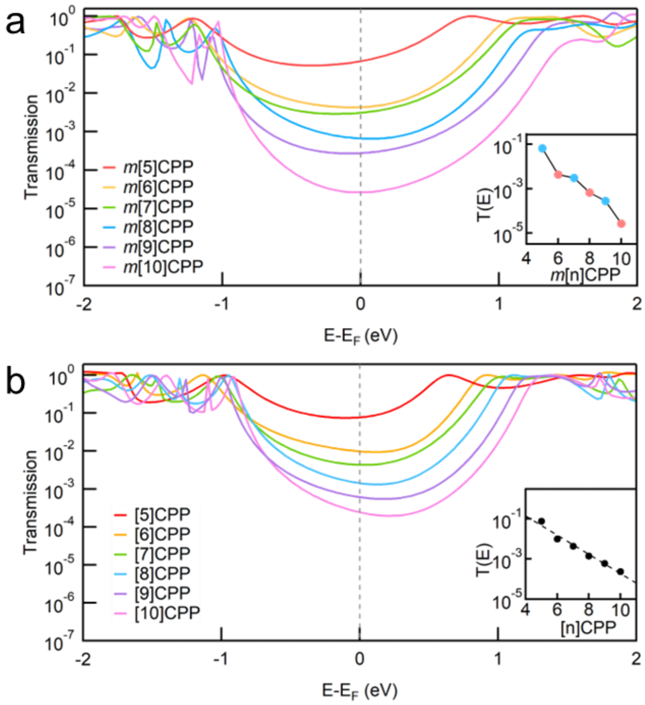

Figure S35 | DFT calculated electron transmission spectra for *m*[n]CPPs and [n]CPPs (n=5-10). (**a**) Transmission spectra of *m*[n]CPPs junctions. Inset: Calculated conductance at the Fermi level as a function of ring size. (**b**) Transmission spectra of [n]CPPs junctions. Inset: Calculated conductance at the Fermi level as a function of ring size. The comparison shows that *m*CPPs display a pronounced odd–even oscillation with increasing ring size, whereas CPPs follow a smooth exponential decay without parity modulation. This trend is fully consistent with the experimental observations.

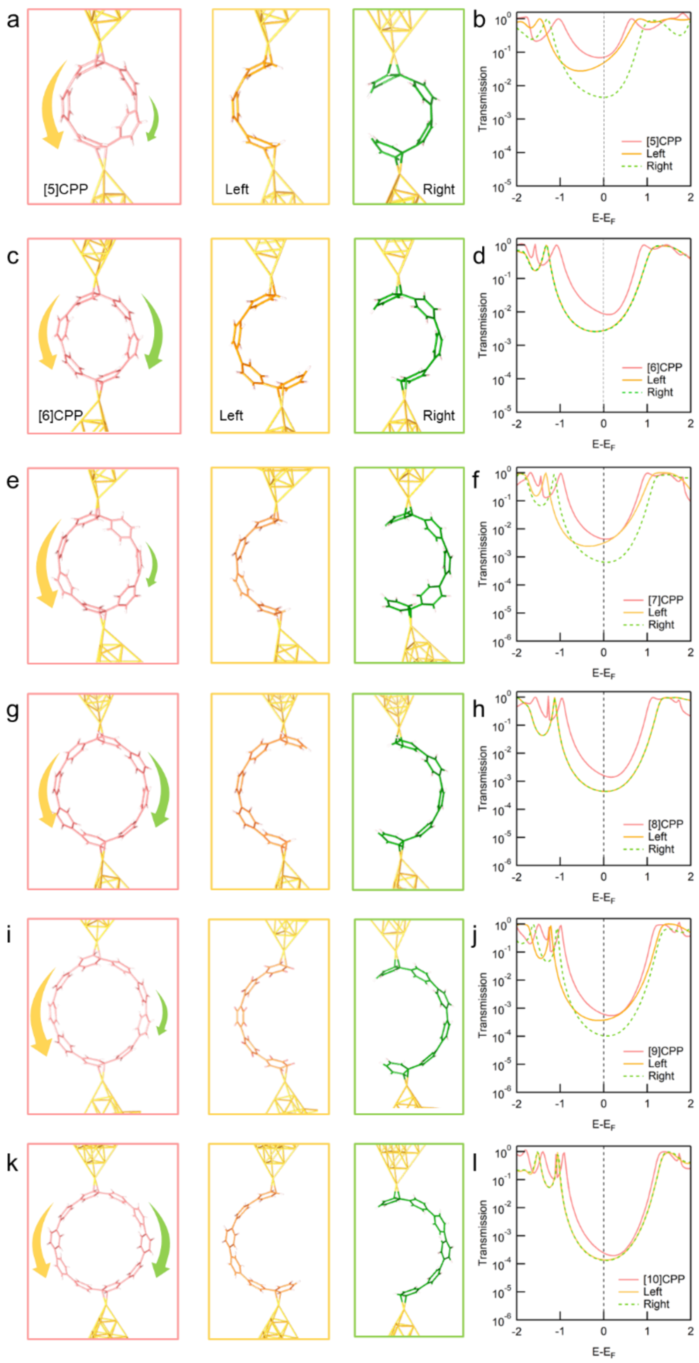

Figure S36 | DFT calculated electron transmission spectra for [n]CPPs (n=5-10) and their individual branches. (**a, c, e, g, i, k**) Junction geometries used for calculating the transmission spectra of the full [n]CPPs (red), left (yellow) and right (green) oligophenylene branches. (**b, d, f, h, j, l**) Corresponding transmission spectra for the full junctions and the two individual branches.

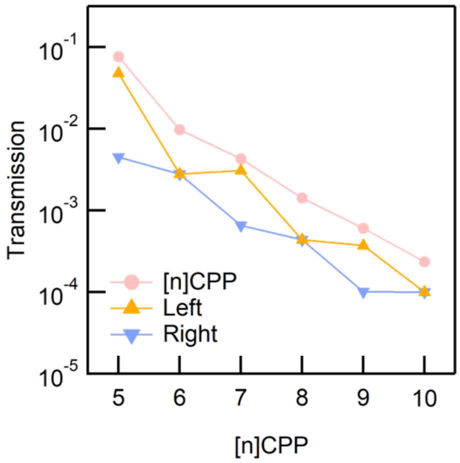

Figure S37 | Branch-resolved conductance analysis of [n]CPPs (n = 5–10). Calculated conductance at the Fermi level for the CPPs and for the isolated left and right pathways. The two pathways show opposite size-dependent trends, and their contributions compensate when combined, yielding a total conductance that follows a smooth exponential decay without an odd–even effect.

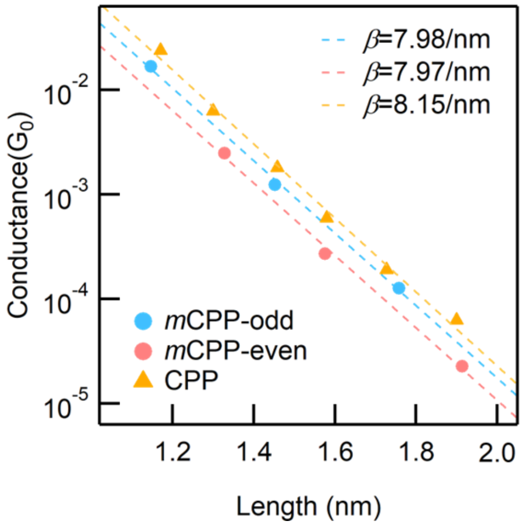

Figure S38 | Decay coefficients of odd- and even-numbered *m*[n]CPPs and [n]CPPs. Length-dependent conductance plots for odd (blue) and even (red) *m*[n]CPPs, as well as [n]CPPs (yellow), with the corresponding linear fits. The extracted decay coefficients are 7.98 nm^{-1} (odd *m*CPPs), 7.97 nm^{-1} (even *m*CPPs), and 8.15 nm^{-1} (CPPs), showing that the introduction of the *meta* unit does not significantly alter the overall tunneling decay.

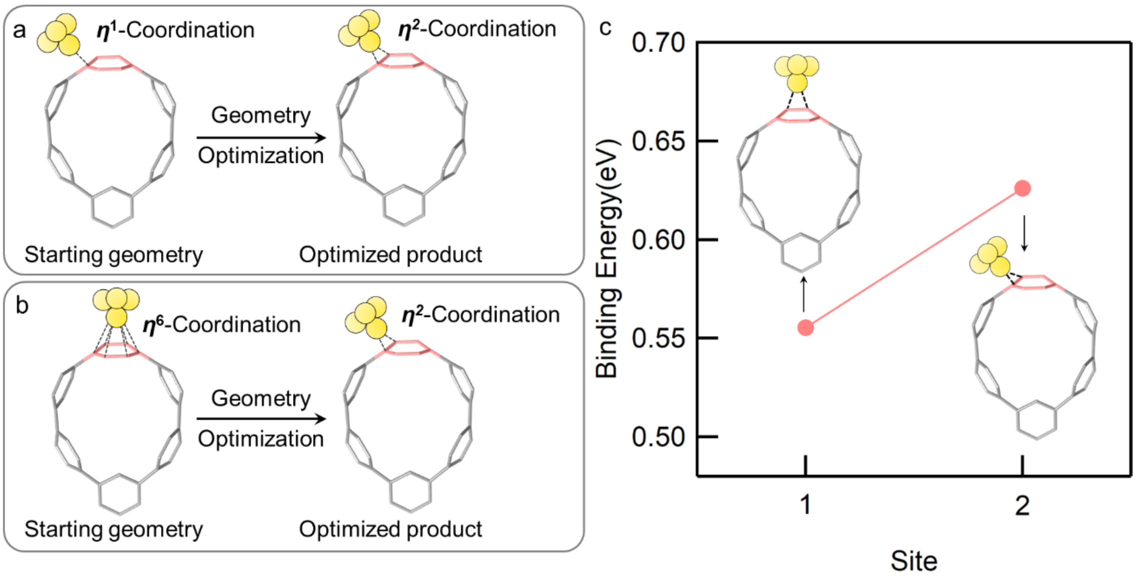

Figure S39 | DFT analysis of Au–π binding motifs within a phenylene unit of *m*[6]CPP. (a, b) Geometry optimizations initiated from η^1 and η^6 Au–phenylene coordination modes both relax to the same η^2 configuration, in which the Au cluster binds to the C=C bond. (**c**) Comparison of binding energies for two intra-ring positions shows that site 2 binds more strongly than site 1, indicating that site 2 is the preferred Au–*π* anchoring location within the phenylene unit.

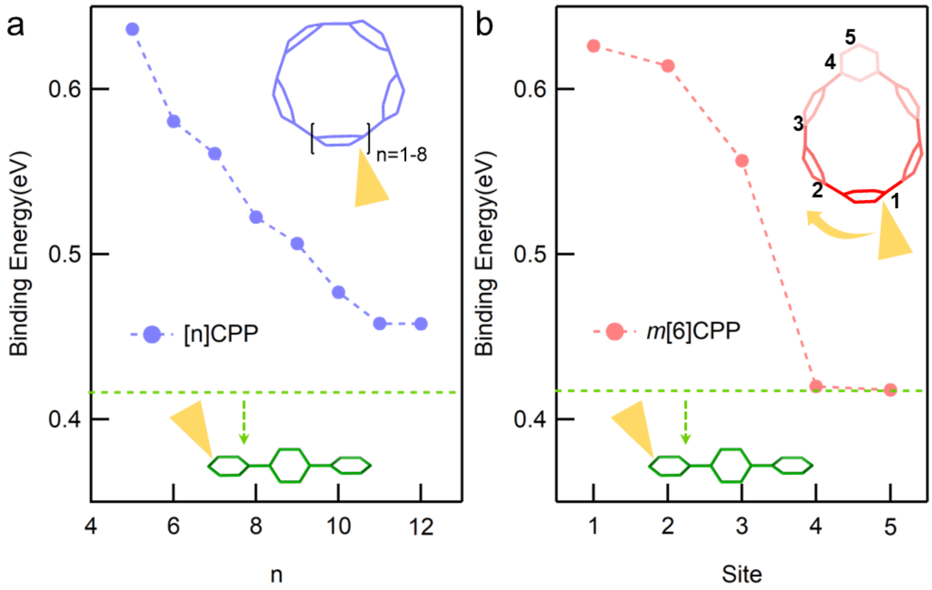

Figure S40 | DFT calculated binding energies for CPP, *m*CPPs and *p*-terphenyl. (**a**) Binding energies of Au coordinated to [n]CPPs of different ring sizes (blue) compared with planar *p*-terphenyl (green dashed line). Inset: electrode–molecule models used in the calculations. (**b**) Binding energies at different intra-ring sites of *m*[6]CPP (red) compared with *p*-terphenyl. Inset: corresponding electrode–molecule models. The curved para-phenylene sites (1–3) show significantly stronger Au–*π* binding than the nearly unstrained *meta*-phenylene

sites (4–5), whose binding energies are similar to planar *p*-terphenyl. This curvature dependence indicates that the *meta* spacer is unlikely to host a stable Au contact.

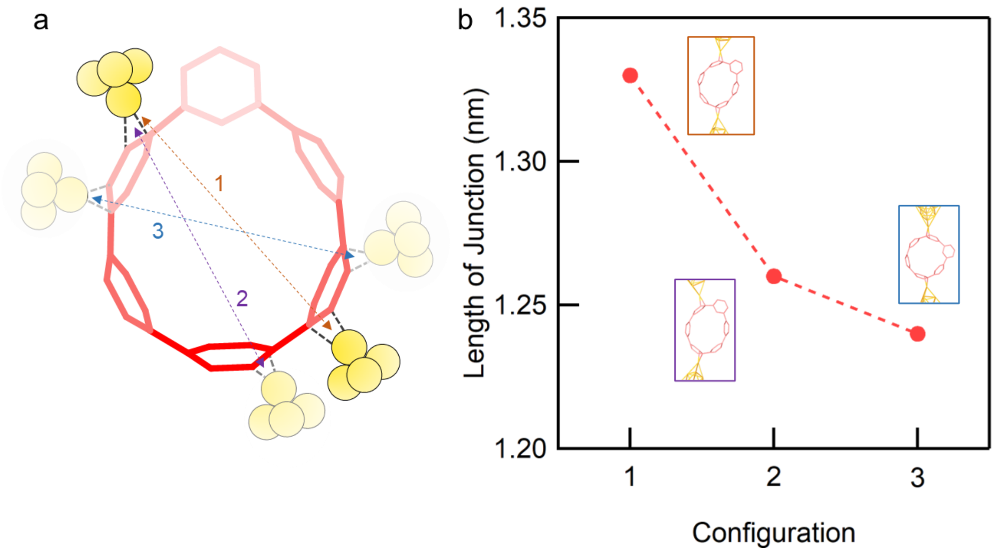

Figure S41 | Junction length analysis for *m*[6]CPP. (**a**) Schematic illustration of Au electrodes binding at different sites of *m*[6]CPP. (**b**) DFT-calculated junction lengths for the three binding configurations shown in (a). Insets: the corresponding DFT-optimized geometries. The configuration-1 configuration produces the longest junction length, which is reached just before junction rupture.

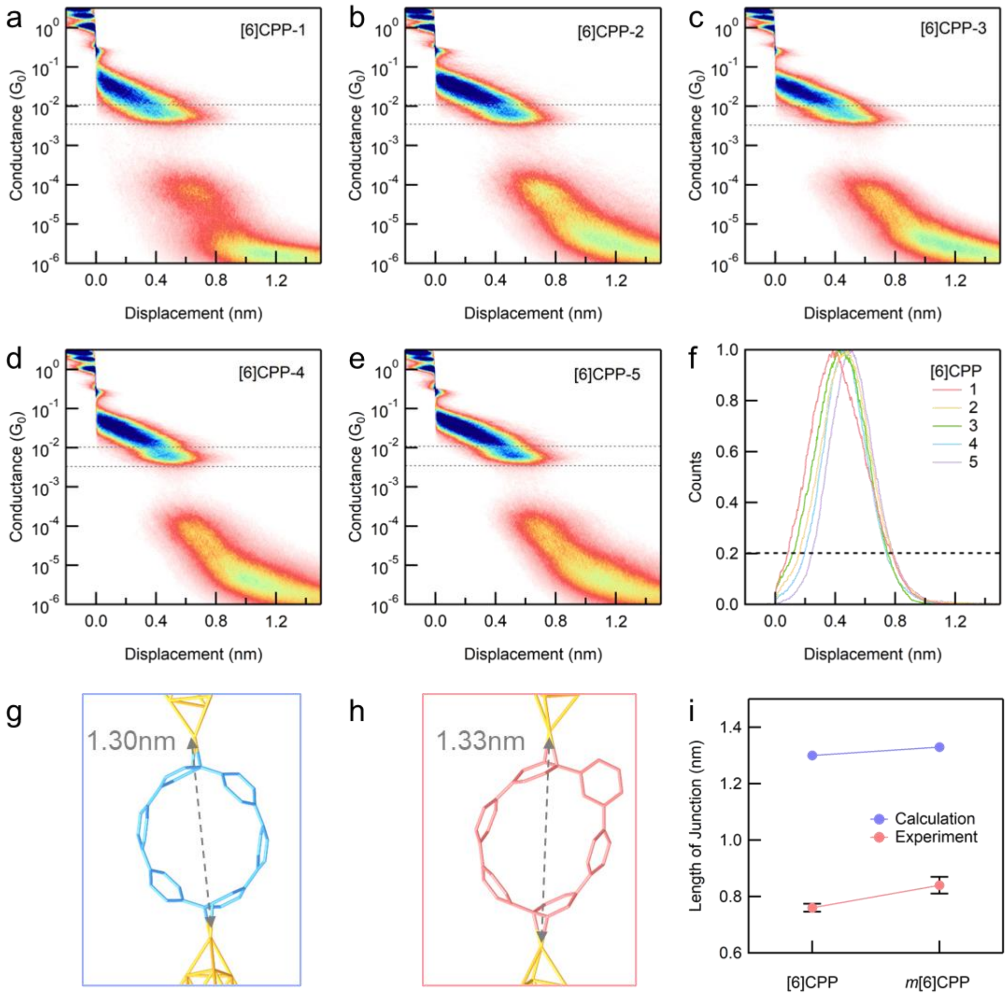

Figure S42 | Junction length analysis of [6]CPP and *m*[6]CPP. (**a-e**) 2D histograms from five independent measurements of [6]CPP. The horizontal dashed lines indicate the integration windows used to profile junction lengths. (**f**) Normalized displacement distributions derived from the 2D histograms. The 80th-percentile displacement values (marked by dashed lines) are used to represent the experimental junction lengths for each [6]CPP. (**g-h**) DFT-calculated junction length comparison between [6]CPP and *m*[6]CPP. (**i**) Comparison of calculated (blue) and experimentally measured (red) junction lengths. Error bars represent the standard deviation. The consistent experimental length difference between *m*[6]CPP and [6]CPP confirms that the junction adopts the longest theoretical path, with configuration 1 as the dominant geometry.

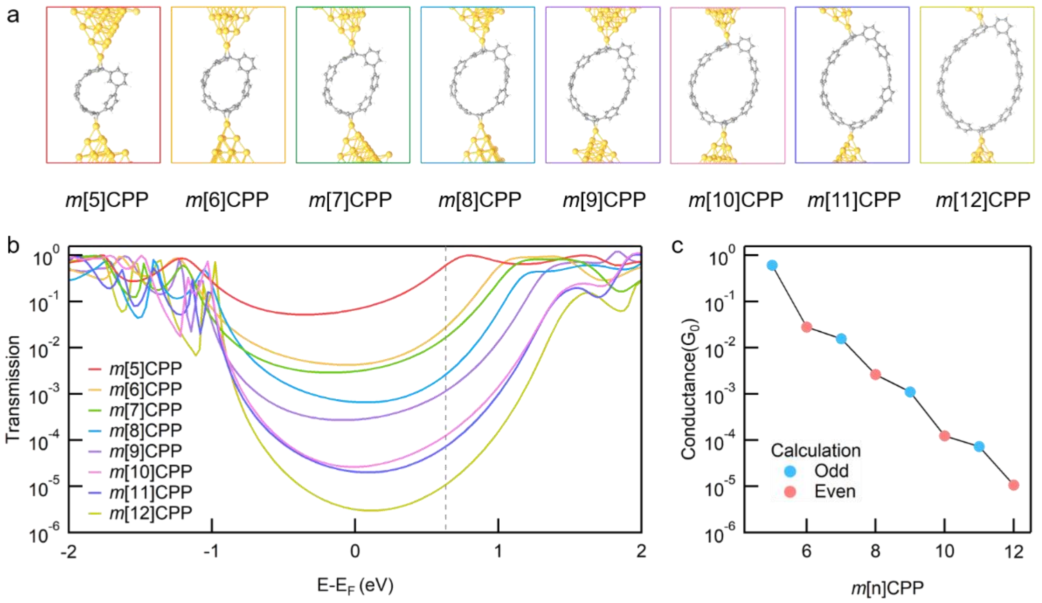

Figure S43 | DFT-based quantum transport properties of *m*[n]CPPs (n = 5-12). (**a**) Geometric structures of *m*[n]CPPs junction geometries (n = 5-12) used in transmission calculations. (**b**) DFT calculated transmission spectra of *m*[n]CPP SMJs ranging from *m*[5]CPP to *m*[12]CPP using the FHI-aims package. (**c**) Calculated conductance values at E_F plotted as a function of phenylene unit in *m*[n]CPPs, derived from the transmission spectra in (**b**). The overall conductance exhibits a pronounced odd–even oscillation with the increasing number of phenylene units in *m*[n]CPP. Blue and red dots denote odd- and even-membered *m*[n]CPPs, respectively, consistent with the color coding used in the main text. This trend agrees well with both the conductance values calculated using SIESTA package in Fig. 5 and those obtained from experimental measurements, thereby validating the accuracy of our conclusions.

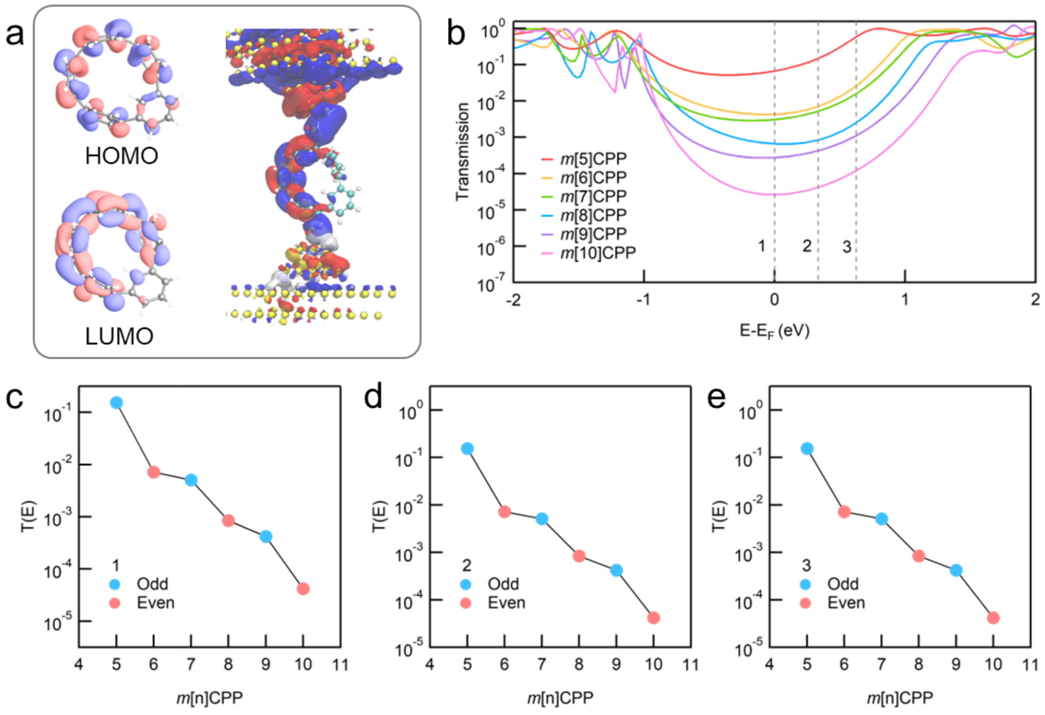

Figure S44 | DFT-based quantum transport properties of *m*[n]CPPs. (**a**) HOMO and LUMO of *m*[5]CPP and the transmission eigenchannel of *m*[5]CPP at the Fermi level (E_F). (**b**) DFT calculated transmission spectra of *m*[n]CPPs SMJs ranging from *m*[5]CPP to *m*[10]CPP using the FHI-aims package. (**c-e**) Calculated conductance values at different E_F plotted as a function of the number of phenylene units in *m*[n]CPPs, derived from the transmission spectra. The calculated conductance of *m*[n]CPPs exhibit an odd-even oscillatory behavior at different Fermi levels.

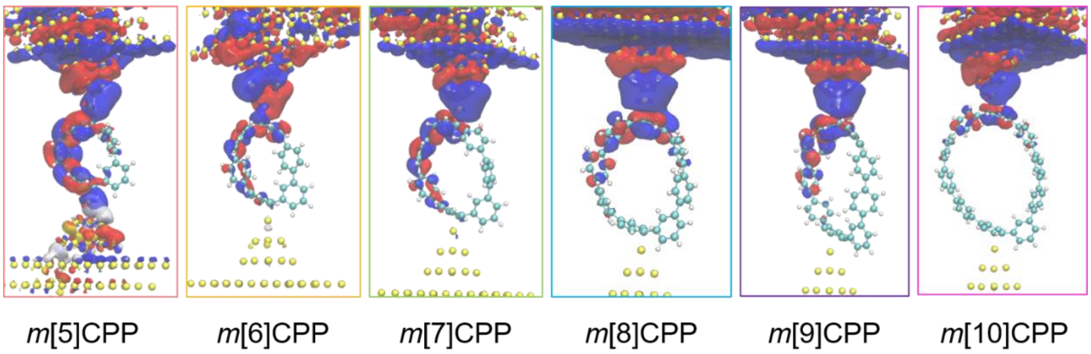

Figure S45 | Spatial distribution of the first transport eigenchannel of the *m*[5]CPP to *m*[10]CPP junction originated from the top electrode.

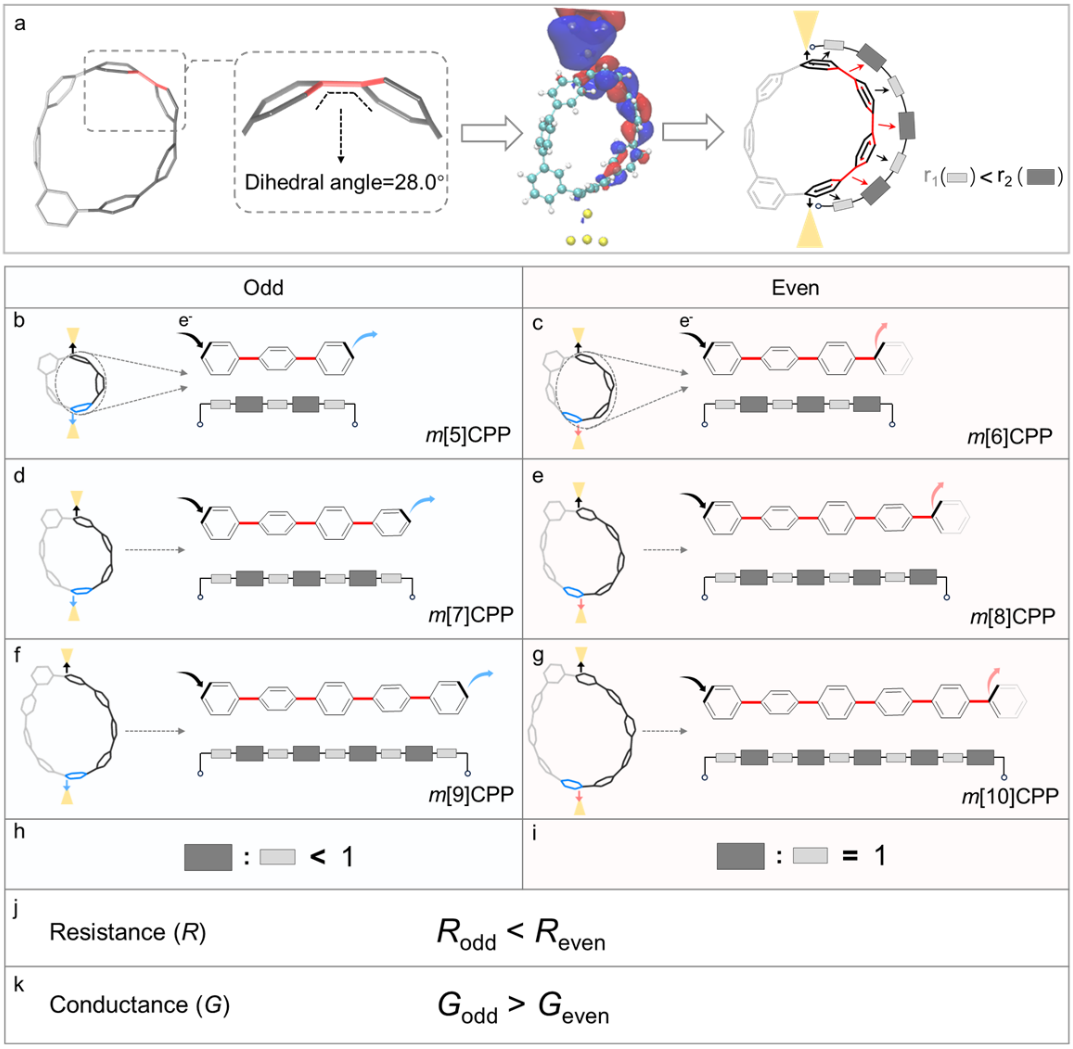

Figure S46 | Equivalent-resistor representation of electron transport in *m*[n]CPP (n = 5–10) junctions. (**a**) DFT-optimized geometry of *m*[7]CPP showing a large inter-ring dihedral angle (~28°), the corresponding first transport eigenchannel, and a simplified series-resistor model in which intra-ring segments (r_1) act as lower-resistance elements and inter-ring segments (r_2) act as higher-resistance elements. (**b, d, f**) Equivalent circuit diagrams for odd-numbered *m*[n]CPPs. (**c, e, g**) Equivalent circuit diagrams for even-numbered *m*[n]CPPs. (**h**) Odd-numbered *m*CPPs sample fewer high-resistance inter-ring segments along the dominant transport path. (**i**) Even-numbered *m*CPPs contain a higher proportion of these high-resistance segments. (**j, k**) As a result, the odd series exhibits a lower overall resistance ($R_{odd} < R_{even}$) and therefore a higher conductance ($G_{odd} > G_{even}$).

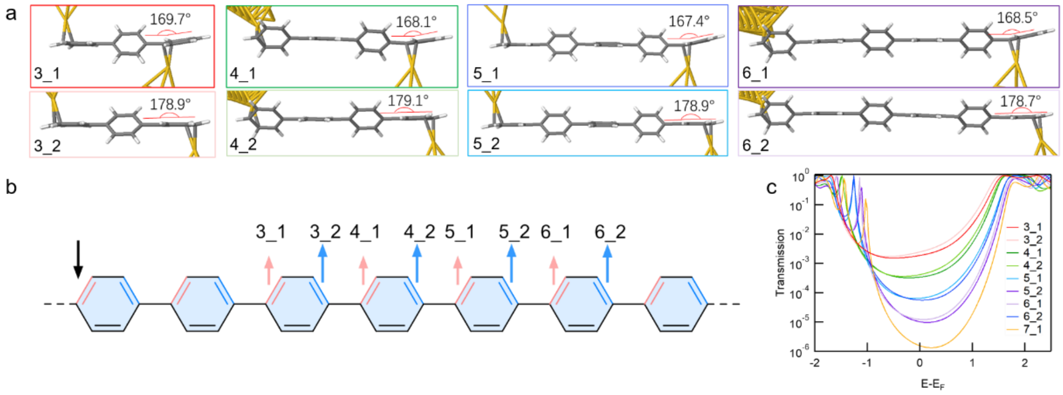

Figure S47 | Even-odd conductance oscillation in linear oligophenylene systems (number of benzene rings varies from 3 to 6). (**a**) DFT-optimized molecular junction geometries (3_1 to 6_2) for transport calculations. Red arcs denote dihedral angles between terminal benzene rings and the molecular backbone plane, highlighting conformational variations. (**b**) Schematic of charge transport pathways in linear oligophenylenes. The black arrow represents the direction of electron injection, and the blue and red arrows represent the directions of electron outflow, respectively. (**c**) Calculated transmission functions of 3_1 to 6_2 by FHI-aims package. The sustained odd-even conductance oscillations observed in planar oligophenylenes provide compelling evidence that this phenomenon originates robustly from orbital topology, independent of molecular curvature or cyclic architecture.

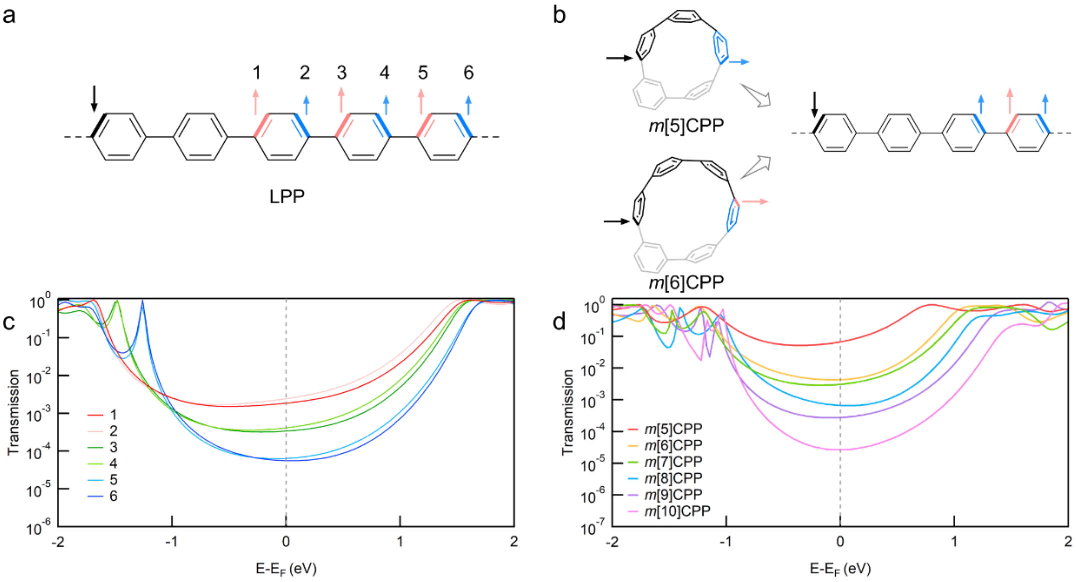

Figure S48 | Even-odd conductance oscillation in linear oligophenylenes (LPPs) and *m*[n]CPPs systems. (**a, b**) Schematic of charge transport pathways in LPPs and *m*[n]CPPs. The black arrow represents the direction of electron injection, and the blue and red arrows represent

the directions of electron outflow, respectively. (**c, d**) DFT calculated transmission spectra of LPPs and *m*[n]CPPs using the FHI-aims package.

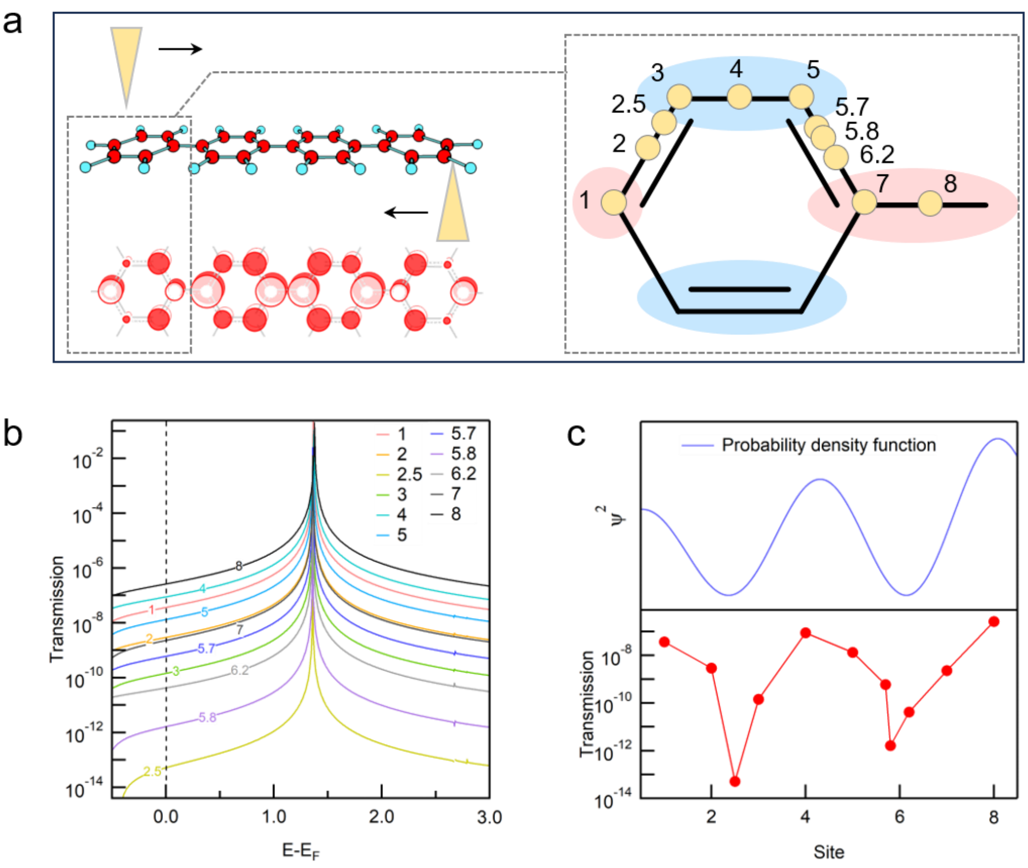

Figure S49 | Transport properties for *para*-tetraphenylene in the level of DFTB model with different contact sites. (**a**) Tight binding calculations based on the tetraphenylene model, where both electrodes simultaneously slide inward along the molecule. The LUMO orbital, calculated via Hückel theory, is shown with its spatial distribution. Zoom-in: Contact sites between the left electrode and the phenylene unit. (**b**) Transmission spectra calculated using a single-orbital (LUMO-only) tight-binding model, highlighting variations in conductance based on the electrode contact sites. (**c**) Comparison between the LUMO orbital's probability density (approximated by a one-dimensional particle-in-a-box model) and the corresponding transmission at the Fermi level (E_F = 0) for different contact sites. The transmission varies systematically with contact sites, closely following the spatial topology of the molecular orbital. These results demonstrate that the spatial distribution of a single molecular orbital can be directly harnessed to modulate quantum transport, offering a strategy to control conductance at the orbital level.

REFERENCES

1. Z. Chen, S. L. Woltering, B. Limburg, M. Y. Tsang, J. Baugh, G. A. D. Briggs, J. A. Mol, H. L. Anderson, J. O. Thomas, Connections to the electrodes control the transport mechanism in single-molecule transistors. *Angew. Chem. Int. Ed. Engl.* **63**, e202401323 (2024).

2. S. V. Aradhya, L. Venkataraman, Single-molecule junctions beyond electronic transport. *Nat. Nanotechnol.* **8**, 399–410 (2013).

3. Z. Chen, J. R. Deng, S. Hou, X. Bian, J. L. Swett, Q. Wu, J. Baugh, L. Bogani, G. A. D. Briggs, J. A. Mol, C. J. Lambert, H. L. Anderson, J. O. Thomas, Phase-coherent charge transport through a porphyrin nanoribbon. *J. Am. Chem. Soc.* **145**, 15265–15274 (2023).

4. A. Feng, Y. Zhou, M. A. Y. Al-Shebami, L. Chen, Z. Pan, W. Xu, S. Zhao, B. Zeng, Z. Xiao, Y. Yang, W. Hong, σ-σ Stacked supramolecular junctions. *Nat. Chem.* **14**, 1158–1164 (2022).

5. C. Jia, A. Migliore, N. Xin, S. Huang, J. Wang, Q. Yang, S. Wang, H. Chen, D. Wang, B. Feng, Z. Liu, G. Zhang, D.-H. Qu, H. Tian, M. A. Ratner, H. Q. Xu, A. Nitzan, X. Guo, Covalently bonded single-molecule junctions with stable and reversible photoswitched conductivity. *Science* **352**, 1443–1445 (2016).

6. Y. S. Park, A. C. Whalley, M. Kamenetska, M. L. Steigerwald, M. S. Hybertsen, C. Nuckolls, L. Venkataraman, Contact chemistry and single-molecule conductance: A comparison of phosphines, methyl sulfides, and amines. *J. Am. Chem. Soc.* **129**, 15768–15769 (2007).

7. M. A. Reed, C. Zhou, C. J. Muller, T. P. Burgin, J. M. Tour, Conductance of a molecular junction. *Science* **278**, 252–254 (1997).

8. L. Venkataraman, J. E. Klare, C. Nuckolls, M. S. Hybertsen, M. L. Steigerwald, Dependence of single-molecule junction conductance on molecular conformation. *Nature* **442**, 904–907 (2006).

9. J. Li, S. Hou, Y. R. Yao, C. Zhang, Q. Wu, H. C. Wang, H. Zhang, X. Liu, C. Tang, M. Wei, W. Xu, Y. Wang, J. Zheng, Z. Pan, L. Kang, J. Liu, J. Shi, Y. Yang, C. J. Lambert, S. Y. Xie, W.

Hong, Room-temperature logic-in-memory operations in single-metallofullerene devices. *Nat. Mater.* **21**, 917–923 (2022).

10. T. A. Su, M. Neupane, M. L. Steigerwald, L. Venkataraman, C. Nuckolls, Chemical principles of single-molecule electronics. *Nat. Rev. Mater.* **1**, 16002 (2016).

11. P. Gehring, J. M. Thijssen, H. S. J. van der Zant, Single-molecule quantum-transport phenomena in break junctions. *Nat. Rev. Phys.* **1**, 381–396 (2019).

12. M. H. Garner, H. Li, Y. Chen, T. A. Su, Z. Shangguan, D. W. Paley, T. Liu, F. Ng, H. Li, S. Xiao, C. Nuckolls, L. Venkataraman, G. C. Solomon, Comprehensive suppression of single-molecule conductance using destructive sigma-interference. *Nature* **558**, 415–419 (2018).

13. J. C. Cuevas, E. Scheer, *Molecular Electronics: An Introduction to Theory and Experiment* (World Scientific, 2010), p. 81.

14. K. Moth-Poulsen, L. Patrone, N. Stuhr-Hansen, J. B. Christensen, J.-P. Bourgoin, T. Bjørnholm, Probing the effects of conjugation path on the electronic transmission through single molecules using scanning tunneling microscopy. *Nano Lett.* **5**, 783–785 (2005).

15. A. R. Garrigues, L. Yuan, L. Wang, E. R. Mucciolo, D. Thompon, E. Del Barco, C. A. Nijhuis, A single-level tunnel model to account for electrical transport through single molecule- and self-assembled monolayer-based junctions. *Sci. Rep.* **6**, 26517 (2016).

16. A. Nitzan, Electron transmission through molecules and molecular interfaces. *Annu. Rev. Phys. Chem.* **52**, 681–750 (2001).

17. Z. L. Cheng, R. Skouta, H. Vazquez, J. R. Widawsky, S. Schneebeli, W. Chen, M. S. Hybertsen, R. Breslow, L. Venkataraman, In situ formation of highly conducting covalent Au-C contacts for single-molecule junctions. *Nat. Nanotechnol.* **6**, 353–357 (2011).

18. Y. Zang, A. Pinkard, Z. F. Liu, J. B. Neaton, M. L. Steigerwald, X. Roy, L. Venkataraman, Electronically transparent Au-N bonds for molecular junctions. *J. Am. Chem. Soc.* **139**, 14845–14848 (2017).

19. K. Yoshizawa, T. Tada, A. Staykov, Orbital views of the electron transport in molecular devices. *J. Am. Chem. Soc.* **130**, 9406–9413 (2008).

20. C. M. Guedon, H. Valkenier, T. Markussen, K. S. Thygesen, J. C. Hummelen, S. J. van der Molen, Observation of quantum interference in molecular charge transport. *Nat. Nanotechnol.* **7**, 305–309 (2012).

21. W. Xu, H. Zhang, Y. Zhou, T. Lu, Y. Li, Y. Zhu, C. Wei, J. Zheng, R. Li, J. Li, L. Chen, G. Zhang, J. Shi, J. Liu, D. Zhang, W. Hong, Supramolecular diodes with donor-acceptor interactions. *J. Am. Chem. Soc.* **147**, 5879–5886 (2025).

22. Y. Zhou, S. Ji, Y. Zhu, H. Liu, J. Wang, Y. Zhang, J. Bai, X. Li, J. Shi, W. Su, R. Huang, J. Liu, W. Hong, Nanoscale evolution of charge transport through C−H⋯π interactions. *J. Am. Chem. Soc.* **146**, 33378–33385 (2024).

23. S. D. Wu, Z. Z. Chen, W. J. Sun, L. Y. Shi, A. K. Shen, J. J. Cao, Z. Liu, C. J. Lambert, H. L. Zhang, Boosting the photoresponse of azobenzene single-molecule junctions via mechanical interlock and dynamic anchor. *ACS Nano* **18**, 31547–31558 (2024).

24. J. E. Greenwald, J. Cameron, N. J. Findlay, T. Fu, S. Gunasekaran, P. J. Skabara, L. Venkataraman, Highly nonlinear transport across single-molecule junctions via destructive quantum interference. *Nat. Nanotechnol.* **16**, 313–317 (2021).

25. R. Frisenda, V. A. Janssen, F. C. Grozema, H. S. van der Zant, N. Renaud, Mechanically controlled quantum interference in individual π-stacked dimers. *Nat. Chem.* **8**, 1099–1104 (2016).

26. M. Zhang, J. Lin, K. Song, K. Chang, X. Dai, Y. Zang, D. Zhu, Iminyl-radical-mediated formation of covalent Au-N bonds for molecular junctions. *J. Am. Chem. Soc.* **145**, 6480–6485 (2023).

27. L. Li, J. Z. Low, J. Wilhelm, G. Liao, S. Gunasekaran, C. R. Prindle, R. L. Starr, D. Golze, C. Nuckolls, M. L. Steigerwald, F. Evers, L. M. Campos, X. Yin, L. Venkataraman, Highly

conducting single-molecule topological insulators based on mono- and di-radical cations. *Nat. Chem.* **14**, 1061–1067 (2022).

28. N. Ferri, N. Algethami, A. Vezzoli, S. Sangtarash, M. McLaughlin, H. Sadeghi, C. J. Lambert, R. J. Nichols, S. J. Higgins, Hemilabile ligands as mechanosensitive electrode contacts for molecular electronics. *Angew. Chem. Int. Ed. Engl.* **58**, 16583–16589 (2019).

29. J. S. Meisner, M. Kamenetska, M. Krikorian, M. L. Steigerwald, L. Venkataraman, C. Nuckolls, A single-molecule potentiometer. *Nano. Lett.* **11**, 1575–1579 (2011).

30. J. N. Gao, A. Bu, Y. Chen, M. Huang, Z. Chen, X. Li, C. H. Tung, L. Z. Wu, H. Cong, Synthesis of all-benzene multi-macrocyclic nanocarbons by post-functionalization of meta-cycloparaphenylenes. *Angew. Chem. Int. Ed. Engl.* **63**, e202408016 (2024).

31. T. C. Lovell, C. E. Colwell, L. N. Zakharov, R. Jasti, Symmetry breaking and the turn-on fluorescence of small, highly strained carbon nanohoops. *Chem. Sci.* **10**, 3786–3790 (2019).

32. R. Jasti, J. Bhattacharjee, J. B. Neaton, C. R. Bertozzi, Synthesis, characterization, and theory of [9]-, [12]-, and [18]cycloparaphenylene: Carbon nanohoop structures. *J. Am. Chem. Soc.* **130**, 17646–17647 (2008).

33. G. Povie, Y. Segawa, T. Nishihara, Y. Miyauchi, K. Itami, Synthesis of a carbon nanobelt. *Science* **356**, 172–175 (2017).

34. Y. Lv, J. Lin, K. Song, X. Song, H. Zang, Y. Zang, D. Zhu, Single cycloparaphenylene molecule devices: Achieving large conductance modulation via tuning radial π-conjugation. *Sci. Adv.* **7**, eabk3095 (2021).

35. J. Lin, Y. Lv, K. Song, X. Song, H. Zang, P. Du, Y. Zang, D. Zhu, Cleavage of non-polar C(sp^2)-C(sp^2) bonds in cycloparaphenylenes via electric field-catalyzed electrophilic aromatic substitution. *Nat. Commun.* **14**, 293 (2023).

36. J. Lin, S. Wang, F. Zhang, B. Yang, P. Du, C. Chen, Y. Zang, D. Zhu, Highly efficient charge transport across carbon nanobelts. *Sci. Adv.* **8**, eade4692 (2022).

37. S. Feng, R. Almughathawi, A. Weber, S. Hou, C. Zhang, J. S. Wossner, B. Esser, C. Lambert, Q. Wu, Y. Li, J. Li, Dual-state ambipolar charge transport in antiaromatic [4]cyclodibenzopentalene single-molecule nanohoops. *J. Am. Chem. Soc.* **147**, 18475–18483 (2025).

38. A. Bu, Y. Zhao, H. Xiao, C. H. Tung, L. Z. Wu, H. Cong, A conjugated covalent template strategy for all-benzene catenane synthesis. *Angew. Chem. Int. Ed. Engl.* **61**, e202209449 (2022).

39. A. I. Yanson, G. R. Bollinger, H. E. van den Brom, N. Agraït, J. M. van Ruitenbeek, Formation and manipulation of a metallic wire of single gold atoms. *Nature* **395**, 783–785 (1998).

40. R. Vardimon, T. Yelin, M. Klionsky, S. Sarkar, A. Biller, L. Kronik, O. Tal, Probing the orbital origin of conductance oscillations in atomic chains. *Nano Lett.* **14**, 2988–2993 (2014).

41. E. G. Emberly, G. Kirczenow, Electron standing-wave formation in atomic wires. *Phys. Rev. B* **60**, 6028–6033 (1999).

42. N. D. Lang, P. Avouris, Oscillatory conductance of carbon-atom wires. *Phys. Rev. Lett.* **81**, 3515–3518 (1998).

43. Y. J. Lee, M. Brandbyge, M. J. Puska, J. Taylor, K. Stokbro, R. M. Nieminen, Electron transport through monovalent atomic wires. *Phys. Rev. B* **69**, 125409 (2004).

44. R. H. Smit, C. Untiedt, G. Rubio-Bollinger, R. C. Segers, J. M. van Ruitenbeek, Observation of a parity oscillation in the conductance of atomic wires. *Phys. Rev. Lett.* **91**, 076805 (2003).

45. H. S. Sim, H. W. Lee, K. J. Chang, Even-odd behavior of conductance in monatomic sodium wires. *Phys. Rev. Lett.* **87**, 096803 (2001).

46. H. Cabrera-Tinoco, A. C. L. Moreira, R. Valencia-Bedregal, L. Borja-Castro, A. Perez-Carreno, A. Lalupu-Garcia, C. Mendoza-Alejo, C. H. W. Barnes, J. W. Seo, L. De Los Santos Valladares, Effective coupling model to treat the odd-even effect on the current-voltage response of saturated linear carbon chains single-molecule junctions. *ACS Omega* **9**, 35323–35331 (2024).

47. S. Wang, M.-Z. Wei, G.-C. Hu, C.-K. Wang, G.-P. Zhang, Mechanisms of the odd-even effect and its reversal in rectifying performance of ferrocenyl-*n*-alkanethiolate molecular diodes. *Org. Electron.* **49**, 76–84 (2017).

48. L. Jiang, C. S. Sangeeth, C. A. Nijhuis, The origin of the odd-even effect in the tunneling rates across EGaIn junctions with self-assembled monolayers (SAMs) of *n*-alkanethiolates. *J. Am. Chem. Soc.* **137**, 10659–10667 (2015).

49. A. Arnold, F. Weigend, F. Evers, Quantum chemistry calculations for molecules coupled to reservoirs: Formalism, implementation, and application to benzenedithiol. *J. Chem. Phys.* **126**, 174101 (2007).

50. J. Wilhelm, M. Walz, M. Stendel, A. Bagrets, F. Evers, Ab initio simulations of scanning-tunneling-microscope images with embedding techniques and application to C58-dimers on Au(111). *Phys. Chem. Chem. Phys.* **15**, 6684–6690 (2013).

51. A. Bagrets, Spin-polarized electron transport across metal-organic molecules: A density functional theory approach. *J. Chem. Theory. Comput.* **9**, 2801–2815 (2013).

52. M. Camarasa-Gómez, D. Hernangómez-Pérez, F. Evers, Spin-orbit torque in single-molecule junctions from ab initio. *J. Phys. Chem. Lett.* **15**, 5747–5753 (2024).

53. M. Camarasa-Gómez, D. Hernangómez-Pérez, J. Wilhelm, A. Bagrets, F. Evers, Molecular transport. arXiv:2411.01680 [cond-mat.mes-hall] (2024).

54. J. P. Perdew, K. Burke, M. Ernzerhof, Generalized gradient approximation made simple. *Phys. Rev. Lett.* **77**, 3865–3868 (1996).

55. V. Blum, R. Gehrke, F. Hanke, P. Havu, V. Havu, X. Ren, K. Reuter, M. Scheffler, Ab initio molecular simulations with numeric atom-centered orbitals. *Comput. Phys. Commun.* **180**, 2175–2196 (2009).

56. V. Havu, V. Blum, P. Havu, M. Scheffler, Efficient integration for all-electron electronic structure calculation using numeric basis functions. *J. Comput. Phys.* **228**, 8367–8379 (2009).

57. J. M. Soler, E. Artacho, J. D. Gale, A. García, J. Junquera, P. Ordejón, D. Sánchez-Portal, The SIESTA method for *ab initio* order-*N* materials simulation. *J. Phys. Condens. Matter* **14**, 2745–2779 (2002).

58. T. Frederiksen, M. Paulsson, M. Brandbyge, A.-P. Jauho, Inelastic transport theory from first principles: Methodology and application to nanoscale devices. *Phys. Rev. B* **75**, 205413 (2007).

59. M. Paulsson, M. Brandbyge, Transmission eigenchannels from nonequilibrium Green's functions. *Phys. Rev. B* **76**, 115117 (2007).

60. M. J. Frisch, G. W. Trucks, H. B. Schlegel, G. E. Scuseria, M. A. Robb, J. R. Cheeseman, G. Scalmani, V. Barone, G. A. Petersson, H. Nakatsuji, X. Li, M. Caricato, A. V. Marenich, J. Bloino, B. G. Janesko, R. Gomperts, B. Mennucci, H. P. Hratchian, J. V. Ortiz, A. F. Izmaylov, J. L. Sonnenberg, D. Williams-Young, F. Ding, F. Lipparini, F. Egidi, J. Goings, B. Peng, A. Petrone, T. Henderson, D. Ranasinghe, V. G. Zakrzewski, J. Gao, N. Rega, G. Zheng, W. Liang, M. Hada, M. Ehara, K. Toyota, R. Fukuda, J. Hasegawa, M. Ishida, T. Nakajima, Y. Honda, O. Kitao, H. Nakai, T. Vreven, K. Throssell, J. A. Montgomery Jr., J. E. Peralta, F. Ogliaro, M. J. Bearpark, J. J. Heyd, E. N. Brothers, K. N. Kudin, V. N. Staroverov, T. A. Keith, R. Kobayashi, J. Normand, K. Raghavachari, A. P. Rendell, J. C. Burant, S. S. Iyengar, J. Tomasi, M. Cossi, J. M. Millam, M. Klene, C. Adamo, R. Cammi, J. W. Ochterski, R. L. Martin, K. Morokuma, O. Farkas, J. B. Foresman, D. J. Fox, Gaussian 16, Revision C.01 (Gaussian Inc., 2016).

61. C. E. Colwell, T. W. Price, T. Stauch, R. Jasti, Strain visualization for strained macrocycles. *Chem. Sci.* **11**, 3923–3930 (2020).

62. J. L. Casals-Sainz, A. Fernandez-Alarcon, E. Francisco, A. Costales, A. Martin Pendas, Bond order densities in real space. *J. Phys. Chem. A* **124**, 339–352 (2020).

63. T. Lu, F. Chen, Multiwfn: A multifunctional wavefunction analyzer. *J. Comput. Chem.* **33**, 580–592 (2012).

64. T. Frauenheim, G. Seifert, M. Elsterner, Z. Hajnal, G. Jungnickel, D. Porezag, S. Suhai, R. Scholz, A self-consistent charge density-functional based tight-binding method for predictive

materials simulations in physics, chemistry and biology. *Phys. Status Solidi B Basic Solid State Phys.* **217**, 41–62 (2000).

65. M. Elstner, D. Porezag, G. Jungnickel, J. Elsner, M. Haugk, T. Frauenheim, S. Suhai, G. Seifert, Self-consistent-charge density-functional tight-binding method for simulations of complex materials properties. *Phys. Rev. B* **58**, 7260–7268 (1998).

66. D. Q. Andrews, G. C. Solomon, R. P. V. Duyne, M. A. Ratner, Single molecule electronics: Increasing dynamic range and switching speed using cross-conjugated species. *J. Am. Chem. Soc.* **130**, 17309–17319 (2008).

67. Y. Zang, E. D. Fung, T. Fu, S. Ray, M. H. Garner, A. Borges, M. L. Steigerwald, S. Patil, G. Solomon, L. Venkataraman, Voltage-induced single-molecule junction planarization. *Nano Lett.* **21**, 673–679 (2021).

68. Y. Zang, Q. Zou, T. Fu, F. Ng, B. Fowler, J. Yang, H. Li, M. L. Steigerwald, C. Nuckolls, L. Venkataraman, Directing isomerization reactions of cumulenes with electric fields. *Nat. Commun.* **10**, 4482 (2019).

69. T. Fu, K. Frommer, C. Nuckolls, L. Venkataraman, Single-molecule junction formation in break-junction measurements. *J. Phys. Chem. Lett.* **12**, 10802–10807 (2021).
